# Challenges for the development of a universal vaccine against leptospirosis revealed by the evaluation of 22 vaccine candidates

**DOI:** 10.3389/fcimb.2022.940966

**Published:** 2022-10-07

**Authors:** Mara A. C. Maia, Everton B. Bettin, Liana N. Barbosa, Natasha R. de Oliveira, Tiffany T. Bunde, Ana Carolina K. Pedra, Guilherme A. Rosa, Elias E. B. da Rosa, Amilton C. P. Seixas Neto, André A. Grassmann, Johnjoe McFadden, Odir A. Dellagostin, Alan J. A. McBride

**Affiliations:** ^1^ Biotechnology Unit, Technological Development Centre, Federal University of Pelotas, Pelotas, Rio Grande do Sul, Brazil; ^2^ Department of Medicine, University of Connecticut Health, Farmington, CT, United States; ^3^ School of Biosciences and Medicine, Faculty of Health and Medical Sciences, University of Surrey, Guildford, United Kingdom

**Keywords:** *Leptospira interrogans*, outer membrane proteins, reverse and structural vaccinology, cell-surface immunoprecipitation, animal model

## Abstract

Leptospirosis is a neglected disease of man and animals that affects nearly half a million people annually and causes considerable economic losses. Current human vaccines are inactivated whole-cell preparations (bacterins) of *Leptospira* spp. that provide strong homologous protection yet fail to induce a cross-protective immune response. Yearly boosters are required, and serious side-effects are frequently reported so the vaccine is licensed for use in humans in only a handful of countries. Novel universal vaccines require identification of conserved surface-exposed epitopes of leptospiral antigens. Outer membrane β-barrel proteins (βb-OMPs) meet these requirements and have been successfully used as vaccines for other diseases. We report the evaluation of 22 constructs containing protein fragments from 33 leptospiral βb-OMPs, previously identified by reverse and structural vaccinology and cell-surface immunoprecipitation. Three-dimensional structures for each leptospiral βb-OMP were predicted by I-TASSER. The surface-exposed epitopes were predicted using NetMHCII 2.2 and BepiPred 2.0. Recombinant constructs containing regions from one or more βb-OMPs were cloned and expressed in *Escherichia coli*. IMAC-purified recombinant proteins were adsorbed to an aluminium hydroxide adjuvant to produce the vaccine formulations. Hamsters (4-6 weeks old) were vaccinated with 2 doses containing 50 – 125 μg of recombinant protein, with a 14-day interval between doses. Immunoprotection was evaluated in the hamster model of leptospirosis against a homologous challenge (10 – 20× ED_50_) with *L. interrogans* serogroup Icterohaemorrhagiae serovar Copenhageni strain Fiocruz L1-130. Of the vaccine formulations, 20/22 were immunogenic and induced significant humoral immune responses (IgG) prior to challenge. Four constructs induced significant protection (100%, *P* < 0.001) and sterilizing immunity in two independent experiments, however, this was not reproducible in subsequent evaluations (0 – 33.3% protection, *P* > 0.05). The lack of reproducibility seen in these challenge experiments and in other reports in the literature, together with the lack of immune correlates and commercially available reagents to characterize the immune response, suggest that the hamster may not be the ideal model for evaluation of leptospirosis vaccines and highlight the need for evaluation of alternative models, such as the mouse.

## Introduction

Leptospirosis is caused by pathogenic spirochetes of the genus *Leptospira* and has a high morbidity in tropical and subtropical countries (Costa et al., 2015; Picardeau, 2017; Baquero and Machado, 2018). The infection is one of the most widespread bacterial zoonosis in the world and is considered a serious public health problem. Humans are accidental and terminal hosts of the pathogen, exhibiting a wide variety of symptoms, ranging from non-specific fever, chills, headache and myalgia to severe leptospirosis, which can manifest as Weil’s disease or leptospirosis-associated pulmonary haemorrhage syndrome (McBride et al., 2005; Croda et al., 2010). Estimates of annual global incidence are of approximately 1 million human cases, but due to the neglected status of the disease this number is likely underestimated (Costa et al., 2015). The disease affects domestic and wild animals, causing economic losses in subsistence and industrial farming (Adler and de la Pena Moctezuma, 2010; Martins and Lilenbaum, 2017). Leptospirosis prevention is essential to reduce the rate of disease and to interrupt the transmission cycle.

Vaccination of human and animal populations in endemic regions is probably the most viable strategy to control the disease (Adler, 2015b; Costa et al., 2015; Grassmann et al., 2017b; Vernel-Pauillac and Werts, 2018). Commercially available whole-cell inactivated vaccines (bacterins) are routinely used in livestock and domestic animals throughout the world (Verma et al., 2013). Although protective against lethal infection, these vaccines elicit an immune response predominantly against leptospiral LPS, a T-independent antigen that induces a short-term immunity and requires annual booster immunizations. In addition, protection elicited by bacterins are restricted to the serovars included in the vaccine preparation. Some 64 different species of *Leptospira* have been described to date, 17 of them are potentially infectious and are classified into over 300 serovars (Adler and de la Pena Moctezuma, 2010; Vincent et al., 2019). Furthermore, bacterins are associated with adverse side-effects in humans, and this has limited their use to high-risk populations in only a few countries (Verma et al., 2013; Grassmann et al., 2017b; Vernel-Pauillac and Werts, 2018; Felix et al., 2020).

The high genomic and phenotypic diversity of pathogenic *Leptospira* spp. is a major drawback for vaccine development (Pereira et al., 2018). The development of a cost-effective vaccine with long-term protection against leptospirosis has been the goal of several research groups around the world, yet it remains elusive (Felix et al., 2020). A universal vaccine against leptospirosis will likely be multivalent, protective against most pathogenic *Leptospira* spp., induce long-term immunity, free of adverse effects and effective for both human and animal use. Most efforts have focused on developing a recombinant vaccine as a substitute for the bacterins (Dellagostin et al., 2017; Grassmann et al., 2017b; Silveira et al., 2017). Alternative approaches based on stimulating the innate immune system have shown interesting results (Potula et al., 2017; Vernel-Pauillac and Werts, 2018; Santecchia et al., 2019). Furthermore, live attenuated mutants have been developed that conferred cross-protective immunity to several pathogenic *Leptospira* spp. and serovars (Srikram et al., 2011; Murray et al., 2018; Wunder et al., 2021).

Reverse and structural vaccinology (RSV) has been successfully used in the design of more effective vaccines for several infectious diseases, reviewed in (Dormitzer et al., 2012; Liljeroos et al., 2015; Rappuoli et al., 2016; Cable et al., 2020). This approach allows a refined protein design for optimization of antigen structure. The application of structural vaccinology (SV) to leptospiral outer membrane proteins (OMPs) has proven useful towards the identification and localization of immunologically accessible epitopes which can bind to MHC-II receptors (Hsieh et al., 2017; Lata et al., 2018). Previously, we carried out a comprehensive bioinformatics analysis based on RSV that identified β-barrel transmembrane proteins (βb-OMPs) in *L. interrogans* (Grassmann et al., 2017a). βb-OMPs are of particular interest as they are integral components of the outer membrane (OM) in diderm bacteria (Schulz, 2002; Wilson and Bernstein, 2016), and usually play an essential role in the survival and successful infection of the host, such as nutrient acquisition and attachment (Adler, 2015a).

In addition, we used cell-surface immunoprecipitation (CSIP) to experimentally identify leptospiral proteins localized on the cell surface of host-adapted *L. interrogans* (Cunha et al., 2017). This immunoproteomics technique was originally developed to identify proteins in *Neisseria meningitidis* using intact meningococcal cells with patient immune sera and identifying precipitated proteins by mass spectrometry (Mendum et al., 2009; Newcombe et al., 2014). This method was successful in identifying protective antigens, including several components of the 4CMenB vaccine (Serruto et al., 2012). Host-adapted leptospires were subjected to CSIP with sera from convalescent leptospirosis patients and the immunoprecipitated proteins were identified by mass spectrometry to detect potentially immunoprotective seroreactive proteins.

In the current study, we report the application of RSV and CSIP for the selection and design of recombinant 22 constructs based on 33 newly described leptospiral OMPs. Structural modelling of these proteins allowed us to predict surface-exposed regions and to identify B-cell and major histocompatibility complex (MHC-II) binding epitopes. Recombinant proteins (individual or combined in chimeras) were evaluated as vaccine candidates in the hamster model of acute leptospirosis.

## Results

### Identification of the 33 leptospiral proteins used as vaccine candidates

Using an RSV approach, our group previously identified 165 putative leptospiral βb-OMPs, representing novel vaccine candidates (Grassmann et al., 2017a). In addition, we adapted the CSIP technique (Mendum et al., 2009; Newcombe et al., 2014) to confirm these findings *in vitro* (Cunha et al., 2017). Using CSIP we identified 157 immunogenic proteins expressed in host-adapted leptospires and recognized by convalescent human patient sera (unpublished data). In the present study, we selected 33 of these proteins for evaluation as 22 novel vaccine candidates against leptospirosis. Of these, six proteins were identified by both RSV and CSIP techniques, and the remaining 27 proteins were identified by RSV (**Table 1**). The selection criteria focused on exposure on the leptospiral cell surface, and was based on their predicted 3D structure and function (**Table 1**). Based on the identities predicted by RSV we selected several OM transporter families, including: eight TonB-dependent receptors (TBDR): LIC10714, LIC10881*, LIC10896*, LIC10964, LIC11268, LIC12374, LIC20151 and LIC20214; three alginate exporters (AlgE): LIC13229, LIC13417*, LIC13477 (*, see below); one orthologue for the COG4313 channel family: LIC11086; and six putative porins: LIC10544, LIC11271, LIC11366, LIC11506 (OmpG), LIC11975, LIC20019. Seven proteins were also predicted to be orthologues of OM efflux proteins (OEPs) and components of the type I secretion system: LIC10496 (TolC), LIC11941, LIC12307, LIC12575, LIC12693, LIC12990, LIC13135; and the leptospiral GspD orthologue LIC11570, a secretin in the type II secretion system. Proteins from other families with essential roles in OM biosynthesis such as the leptospiral LptD orthologue (LIC11458); a protein that is part of the subfamily of the FadL fatty acid transporter family (LIC11211); and five proteins from the Omp85 family: LIC10539, LIC11623 (BamA), LIC12252, LIC12254 and LIC12258 were also included.

**Table 1:** Closest PDB structures and scores used to predict the 3D models of the β-barrel OMPs.

Gene ID	Closest PDB structure, organism of origin^a^	Identificationmethod	Predictedfamily	PDB	TM-score	RMSD	IDEN	CoV
**LIC10496**	Outer membrane protein TolC, *E. coli*	RSV/CSIP	OEP	1tqqA	0.87	0.94	0.14	0.88
**LIC10539**	Membrane transporter protein FhaC, *Bordetella pertussis* Tohama I	RSV/CSIP	Omp85	4qky	0.94	0.69	0.07	0.95
**LIC10544**	Outer membrane porin OprO, *Pseudomonas aeruginosa*	RSV	Porin	4rjwA	0.77	1.93	0.09	0.80
**LIC10714**	Ferrichrome-iron receptor FhuA, *E. coli*	RSV	TBDR	1fi1A	0.84	0.83	0.17	0.85
LIC10881*	Ferredoxin receptor FusA, *Pectobacterium atrosepticum*	RSV/CSIP	TBDR	4zgvA	0.77	1.93	0.12	0.78
LIC10896*	Ferripyoverdine receptor, *Pseudomonas aeruginosa*	RSV	TBDR	2w16A	0.72	1.97	0.12	0.74
**LIC10964**	Outer membrane transporter ZnuD, *Neisseria meningitidis*	RSV	TBDR	4rdrA	0.84	1.14	0.20	0.85
**LIC11086**	Outer membrane channel COG4313 protein, *Pseudomonas putida* F1	RSV/CSIP	Channel	4rl8A	0.78	1.06	0.13	0.80
**LIC11211**	Outer membrane protein Tbux, *Ralstonia pickettii*	RSV	FadL	3bryB	0.88	2.20	0.09	0.96
**LIC11268**	Transferrin-binding protein A, *Neisseria meningitidis* serogroup B	RSV	TBDR	3v89A	0.98	1.24	0.09	1.00
**LIC11271**	Outer membrane protein W, *E. coli*	RSV	Porin	2f1tA	0.59	2.34	0.09	0.65
**LIC11366**	Outer membrane protease plasminogen activator Pla, *Yersinia pestis*	RSV	Porin	2x4mA	0.63	4.13	0.08	0.82
**LIC11458**	Lipopolysaccharide assembly protein LptD, *Shigella flexneri*	RSV	LptD	4q35A	0.73	1.21	0.15	0.74
**LIC11506**	Outer membrane protein OmpG, *E. coli*	RSV	Porin	2x9kA	0.66	3.29	0.11	0.78
**LIC11570**	Type II secretion system protein GspD, *Vibrio cholerae*	RSV/CSIP	GspD	5wq8A	0.68	2.29	0.27	0.71
**LIC11623**	β-barrel membrane protein BamA, *Neisseria gonorrhoeae*	RSV	BamA	4k3bA	0.79	0.91	0.20	0.79
**LIC11941**	Outer membrane channel CmeC, *Campylobacter jejuni*	RSV	OEP	4mt4A	0.87	1.97	0.12	0.92
**LIC11975**	Autotransporter adhesin AIDA-I, *E. coli*	RSV	Porin	4meeA	0.81	1.56	0.12	0.85
**LIC12252**	β-barrel membrane protein BamA, *Haemophilus ducreyi*	RSV	BamA	4k3cA	0.88	1.19	0.12	0.89
**LIC12254**	β-barrel membrane protein BamA, *Haemophilus ducreyi*	RSV	BamA	4k3cA	0.86	1.51	0.14	0.88
**LIC12258**	β-barrel membrane protein BamA, *Haemophilus ducreyi*	RSV	BamA	4k3cA	0.92	1.70	0.15	0.96
**LIC12307**	Outer membrane protein OprM, *Pseudomonas aeruginosa*	RSV	OEP	1wp1A	0.88	2.27	0.13	0.93
**LIC12374**	Outer membrane receptor HasR, *Serratia marcescens*	RSV	TBDR	3cslB	0.84	3.42	0.14	0.92
**LIC12575**	Outer membrane protein TolC, *E. coli*	RSV	OEP	1tqqA	0.85	0.91	0.14	0.86
**LIC12693**	Outer membrane protein TolC, *E. coli*	RSV	OEP	1tqqA	0.79	1.31	0.14	0.81
**LIC12990**	Outer membrane protein OprM, *Pseudomonas aeruginosa*	RSV	OEP	1wp1A	0.85	2.56	0.11	0.91
**LIC13135**	Outer membrane channel CmeC, *Campylobacter jejuni*	RSV	OEP	4mt4A	0.66	0.68	0.15	0.66
**LIC13229**	Outer membrane porin AlgE, *Pseudomonas aeruginosa*	RSV	AlgE	4afkA	0.71	1.29	0.14	0.72
LIC13417*	Outer membrane porin AlgE, *Pseudomonas aeruginosa*	RSV/CSIP	AlgE	3rbhA	0.66	1.79	0.09	1.00
**LIC13477**	Outer membrane porin AlgE, *Pseudomonas aeruginosa*	RSV	AlgE	4afkA	0.83	1.72	0.14	0.87
**LIC20019**	Outer membrane protease plasminogen activator Pla, *Yersinia pestis*	RSV	Porin	2x4mA	0.78	2.10	0.13	0.83
**LIC20151**	Outer membrane vitamin B12 transporter BtuB, *E. coli*	RSV	TBDR	2gskA	0.80	1.09	0.21	0.81
**LIC20214**	TonB-dependent receptor, siderophore PirA, *Acinetobacter baumanni*	RSV	TBDR	5fr8A	0.78	1.44	0.13	0.80

a As determined by I-TASSER.

Multiple sequence alignments of LIC13417 and its orthologues strongly suggested that it was likely to be a truncated protein, similar to our previous observations for LIC10881* and LIC10896* (Grassmann et al., 2017a). An analysis of the LIC13417 locus in the *L. interrogans* Fiocruz L1-130 genome, revealed the presence of a potentially erroneous stop codon in the last nucleotide of LIC13417. When the point mutation was altered to a serine codon (TAG → TCG), we reassembled LIC13417 and LIC13418 as a single CDS, named hereafter as LIC13417*. A multiple sequence alignment of LIC13417* and its orthologues showed an alignment with 80-97% amino acid identity (AAI) over the full-length of the modified protein (**Figure S1** in **Supplementary Material**).

### Conservation of the vaccine candidates among the pathogenic *Leptospira* spp.

The level of conservation of the vaccine candidates among nine pathogenic *Leptospira* spp. was evaluated by multiple sequence alignment. The alignments were performed using the full-length amino acid sequences of the proteins from the *L. interrogans* Fiocruz L1-130 genome and their orthologues in nine additional pathogenic species (**Figure S2** in **Supplementary Material**). Overall, the proteins showed high levels of conservation; the AAIs ranged from 60.4 – 99.0% among the pathogenic species. Of the selected proteins, 32/33 had orthologues among the eight species that belong to node 1 in the P1 subclade of pathogenic *Leptospira* spp. (Vincent et al., 2019). While LIC10496 was the least conserved protein, it was only found in *L. interrogans*, *L. kirschneri* and *L. noguchii*, these species are generally recognised as the most virulent species of the P1 subclade, and most often associated with severe leptospirosis in humans. The βb-OMPs in these three species were highly conserved, with AAIs > 90% in 31/33 of the vaccine targets. LIC10496 (TolC) and LIC11506 (OmpG) were the least conserved, while those with essential roles in metabolism, such as LIC11623 (BamA), LIC11570 (GspD) and LIC11458 (LptD), demonstrated high AAI (88.6 – 99.0%) among the genomes analysed.

### Epitope prediction in the surface exposed regions of the vaccine candidates

As opsonophagocytosis likely plays an important role in the clearance of leptospires during an infection, we predicted the immunogenic potential of the selected βb-OMPs to induce T- and B-cell responses. NetMHCII 2.2 server and BepiPred 2.0 were used to predict epitopes that could bind to MHC class II molecules encoded by 51 HLA-DRB alleles and linear B-cell epitopes, respectively. The 3D models of each βb-OMPs were aligned with their corresponding analogues, as indicated by I-TASSER (**Table 1**). All the βb-OMPs were predicted to contain surface-exposed linear B-cell epitopes. T-cell epitopes to all 51 of the HLA-DRB alleles were identified in surface-exposed regions in all the βb-OMPs apart from LIC13229, which did not contain epitopes for the DRB1_1502 allele. The list of the T- and B-cell epitopes identified for each βb-OMP is provided (**Table S1** in **Supplementary Material**).

### Construction of leptospiral βb-OMP vaccine candidates

**Figure 1:**
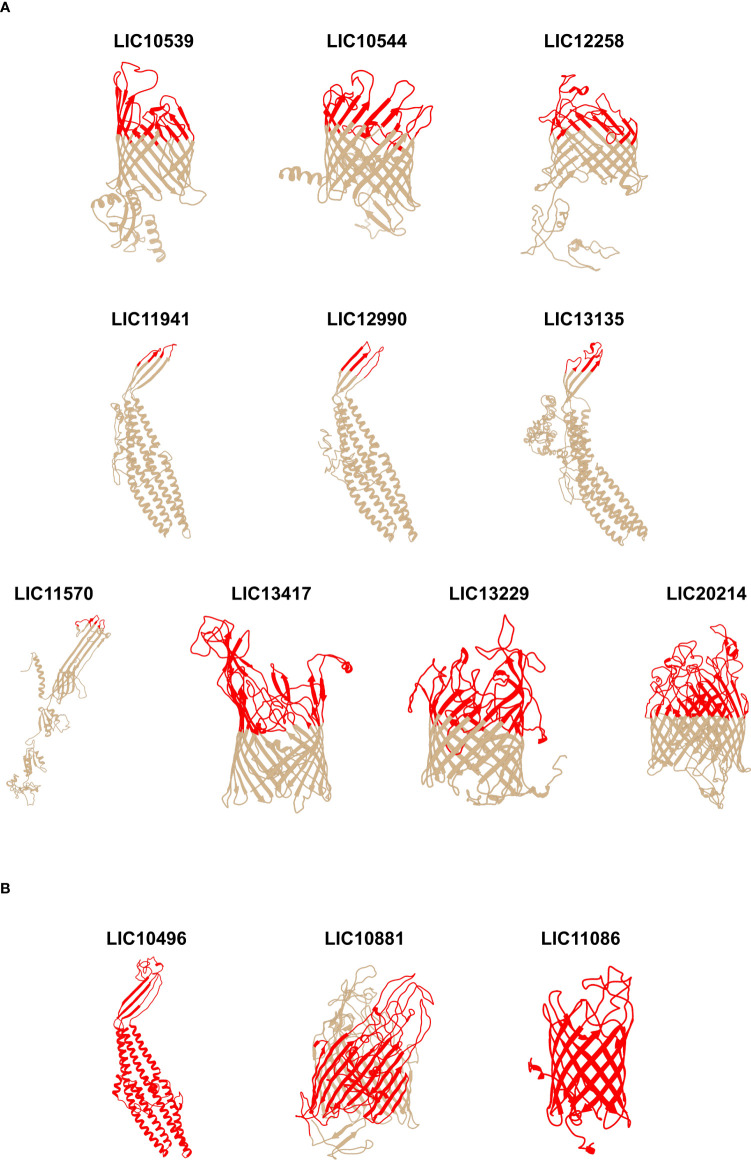
Predicted 3D models of the individual leptospiral β-barrel OMPs and mapping of the surface-exposed regions. The 3D models were determined using I-TASSER and the images were generated using UCSF Chimera software. **(A)** surface-exposed regions of 10 βb-OMPs (constructs do not contain transmembrane or periplasmic regions); **(B)** full-length protein constructs. The orientation of the βb-OMPs in the OM was derived from an interpretation of the best matching PDB structure. The upper, surface-exposed regions are shown in colour on the 3D models and these regions were used in the design of the recombinant proteins. The horizontal bars under the 3D models are a graphical representation of the composition of the chimeras.

**Figure 2:**
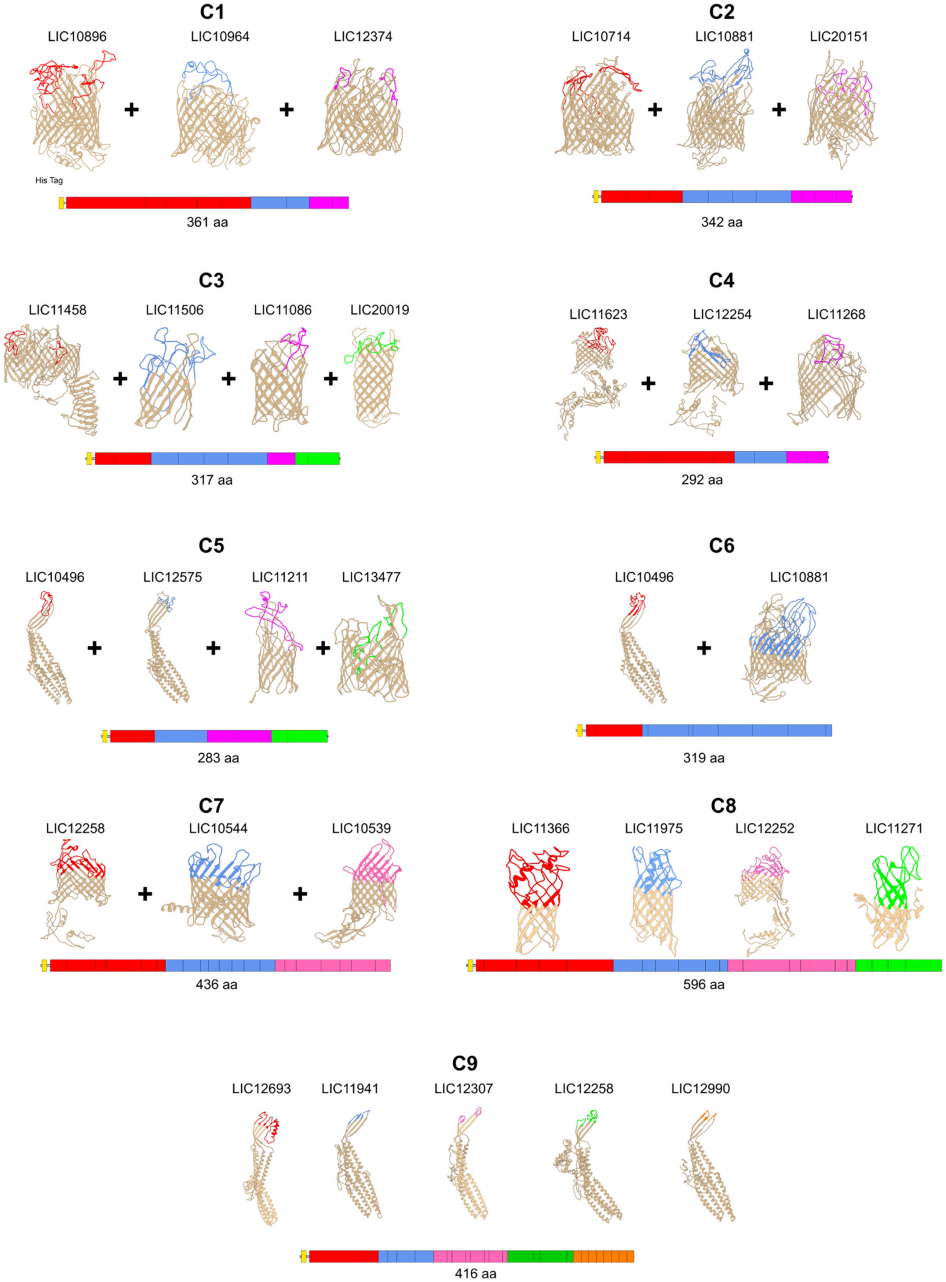
Predicted 3D models of the chimeric leptospiral β-barrel OMPs and mapping of the surface-exposed regions. Chimeras containing combinations of surface-exposed regions are shown. The 3D models were determined using I-TASSER and the images were generated using UCSF Chimera software. The orientation of the βb-OMPs in the OM was derived from an interpretation of the best matching PDB structure. The upper, surface-exposed regions are shown in colour on the 3D models and these regions were used in the design of the recombinant proteins. The horizontal bars under the 3D models are a graphical representation of the composition of the chimeras.

**Figure 3:**
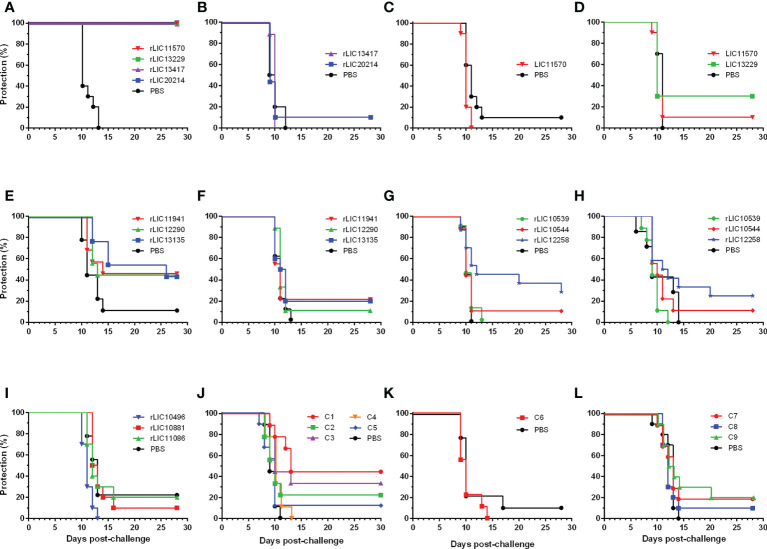
Protection against lethal challenge in the hamster model of acute leptospirosis. Representative experiments showing survival of hamsters vaccinated (days -28 and -14) with two doses of the rβb-OMPs vaccine candidates or injected with a PBS control, followed by challenge (day 0) with a lethal dose of *L. interrogans* serovar Copenhageni strain Fiocruz L1-130, see (**Table 2**). Groups of hamsters were vaccinated with: **(A)** rLIC11570 (GspD), rLIC13229 (AlgE), rLIC13417* (AlgE), rLIC20214 (TBDR) or the PBS control. All the rβb-OMPs induced 100% protection (*P* < 0.0001) and sterilizing immunity; **(B–D)** repeat experiments failed to reproduce the protective immune response seen previously; **(E)** vaccinated with the OEPs rLIC11941, rLIC12290, rLIC13135 or the PBS control. While the level of protection was the same (44.4%) for all proteins, only rLIC13135 significantly increased survival (*P* < 0.05) among vaccinated hamsters compared to the PBS control group; **(F)** a repeat experiment using the OEPs failed to reproduce these results; **(G, H)** vaccinated with rLIC10539 (Omp85), rLIC10544 (porin), rLIC12258 (Omp85) or the PBS control. None of the vaccine preparations protected against the challenge dose; **(I)**, vaccinated with rLIC10496 (TolC), rLIC10881* (TBDR), rLIC11086 (AlgE) or the PBS control. None of the vaccine preparations protected against the challenge dose; **(J)**, hamsters were vaccinated with chimeras C1-C5 or the PBS control. While none of the chimeras protected against the lethal challenge, chimeras C1 (TBDRs: LIC10896* + LIC10964 + LIC12374) and C3 (LIC11458 (LptD) + LIC11506 (OmpG) + LIC11086 (OM channel) + LIC20019 (porin)) significantly increased survival compared to the PBS control group; **(K, L)**, hamsters vaccinated with chimeras C6-C9 or the PBS control group. None of the chimeras induced a significant protective immune response.

Using the data from the structure and function analysis together with the epitope-mapping results, we constructed 22 recombinant proteins containing surface-exposed regions from the 33 leptospiral βb-OMPs for evaluation as vaccine candidates. Three different cloning strategies were used: 1) surface-exposed regions, n = 10 (**Figure 1A**); 2) full-length proteins, n = 3 (**Figure 1B**); 3) chimeras containing combinations of surface-exposed regions from 31 βb-OMPs, n = 9 (**Figure 2**). Details of the 22 recombinant proteins and the 33 βb-OMPs used in their construction are provided (**Table S2** in **Supplementary Material**). The recombinant proteins were characterised by immunoblotting with an anti-His antibody, see (**Figure S3** in **Supplementary Material**).

### Evaluation of βb-OMP vaccine candidates against lethal leptospirosis

In total, thirteen experiments were performed to determine the efficacy of 22 vaccine candidates that were based on 33 βb-OMPs identified by RSV and CSIP, (**Figure 3** and **Table 2**). The vaccination scheme used two doses that ranged from 50 – 125 μg total recombinant protein, adsorbed to an aluminium hydroxide adjuvant and administered intramuscularly. The endpoint for 50% (ED_50_) of infected hamsters was calculated as described previously (Conrad et al., 2017) and was approximately 5 leptospires for the *L. interrogans* Fiocruz L1-130 challenge strain. In two independent experiments, rLIC11570 (GspD), rLIC13229 (AlgE), rLIC13417* (AlgE) and rLIC20214 (TBDR) conferred significant protection in 100% of vaccinated animals (*P* < 0.0001), see (**Figure 3A** and **Table 2**). However, when we re-evaluated these vaccine candidates in an additional three independent experiments, they failed to induce a significant protective immune response; protection ranged from 0 – 30% (**Figures 2B–D** and **Table 2**). Endpoint criteria were observed in the control and vaccinated groups from days 9 – 13 post-challenge (PC), these animals were immediately euthanized, and all survivors were euthanized on day 28. Among the OEPs, only rLIC13135 significantly increased survival (Log-rank, *P* < 0.05) among vaccinated hamsters, (**Figure 3E**; **Table 2**). For LIC11941 and LIC12990, while the level of protection was the same as LIC13135 (44.4%), these results were not significant in terms of mortality or survival. An additional experiment failed to improve on these initial findings (**Figure 3F**; **Table 2**). Hamsters exhibited endpoint criteria on days 10 – 15 PC. LIC10539 (Omp85), LIC10544 (porin) and LIC12258 (Omp85) failed to induce significant protection in vaccinated animals, which ranged from 0 – 22.2% (**Figures 3G–H**; **Table 2**). Most endpoint criteria were observed on days 9 – 14 PC. For the remaining βb-OMPs, LIC10496 (TolC), LIC10881* (TBDR) and LIC11086 (AlgE), protection was not significant; ranging from 0 – 20%, see (**Figure 3I**; **Table 2**). Hamsters exhibited endpoint criteria on days 10 – 14 PC. Vaccine preparations based on the chimera constructs (C1 – C9) contained 25 μg for each protein in the chimera; each dose ranged from 50 – 125 μg total recombinant protein. In three independent experiments, most of the chimeras failed to induce significant protective responses, which ranged from 0 – 22.2%, see (**Figures 3J–L**; **Table 2**). However, vaccination with chimeras C1 (TBDRs: LIC10896* + LIC10964 + LIC12374) and C3 (LIC11458 (LptD) + LIC11506 (OmpG) + LIC11086 (OM channel) + LIC20019 (porin)) resulted in increased survival compared to the control group (Log-rank, *P* < 0.05), see (**Figure 3I**; **Table 2**). Hamsters exhibiting endpoint criteria were observed on days 7 – 20 PC.

**Table 2:** Evaluation of the vaccine candidates in the hamster model of acute leptospirosis.

Vaccine	Dose (μg)	Challenge(ED_50_)	Protection (%)^a^				
**LIC11570**	50/50	10×	100 (10/10)⁑	100 (10/10)⁑		0 (0/10)	10.0 (1/10)
**LIC13229**	50/50	10×	100 (10/10)⁑	100 (10/10)⁑			30.0 (3/10)
LIC13417*	50/50	10×	100 (10/10)⁑	100 (10/10)⁑	0 (0/9)		
**LIC20214**	50/50	10×	100 (10/10)⁑	100 (10/10)⁑	11.1 (1/9)		
**Control**		10×	0 (0/10)	0 (0/10)	0 (0/9)	10.0 (1/10)	0 (0/10)
**LIC11941**	50/50	10×	44.4 (4/9)	22.2 (2/9)			
**LIC12990**	50/50	10×	44.4 (4/9)	11.1 (1/9)			
**LIC13135**	50/50	10×	44.4 (4/9)^†^	11.1 (1/9)			
**Control**		10×	11.1 (1/9)	0 (0/9)			
**LIC10539**	50/50	10×	0 (0/9)	0 (0/9)			
**LIC10544**	50/50	10×	11.1 (1/9)	11.1 (1/9)			
**LIC12258**	50/50	10×	11.1 (1/9)	22.2 (2/9)			
**Control**		10×	0 (0/9)	0 (0/9)			
**LIC10496**	50/50	10×	0 (0/10)				
LIC10881*	50/50	10×	10.0 (1/10)				
**LIC11086**	50/50	10×	20.0 (2/10)				
**Control**		10×	20.0 (2/10)				
C1^b^	50/50	20×	44.4 (4/9)^†^				
**C2**	50/50	20×	22.2 (2/9)				
**C3**	50/50	20×	33.3 (3/9)^†^				
**C4**	50/50	20×	0 (0/9)				
**C5**	50/50	20×	11.1 (1/9)				
**Control**		20×	0 (0/9)				
**C6**	50/50	10×	0 (0/10)				
**Control**		10×	10.0 (1/10)				
**C7**	75/75	10×	20.0 (2/10)				
**C8**	100/100	10×	10.0 (1/10)				
**C9**	125/125	10×	20.0 (2/10)				
**Control**		10×	0 (0/10)				

⁑ Significant protection induced (P < 0.001), otherwise not significant (Fisher exact test), and sterilizing immunity (100%) as determined by culture isolation and qPCR.

^†^ Significantly increased survival (P < 0.05), Log-rank test.

a Protection (%), the number of survivors/total are shown in parentheses, in 13 independent experiments with matched controls.

b Chimera constructs, C1: LIC10896*+LIC10964+LIC12374; C2: LIC10714+LIC10881*+LIC20151; C3: LIC11458+LIC11506+LIC11086+LIC20019; C4: LIC11623+LIC12254+LIC11268; C5: LIC10496+LIC12575+LIC11211+LIC13417*; C6: LIC10496+LIC10881*; C7: LIC10539+LIC10544+LIC12258; C8: LIC11271+LIC11366+LIC11975+LIC12252; C9: LIC11941+LIC12290+LIC13135+LIC12693+LIC12307.

### Humoral immune response in vaccinated hamsters

**Figure 4:**
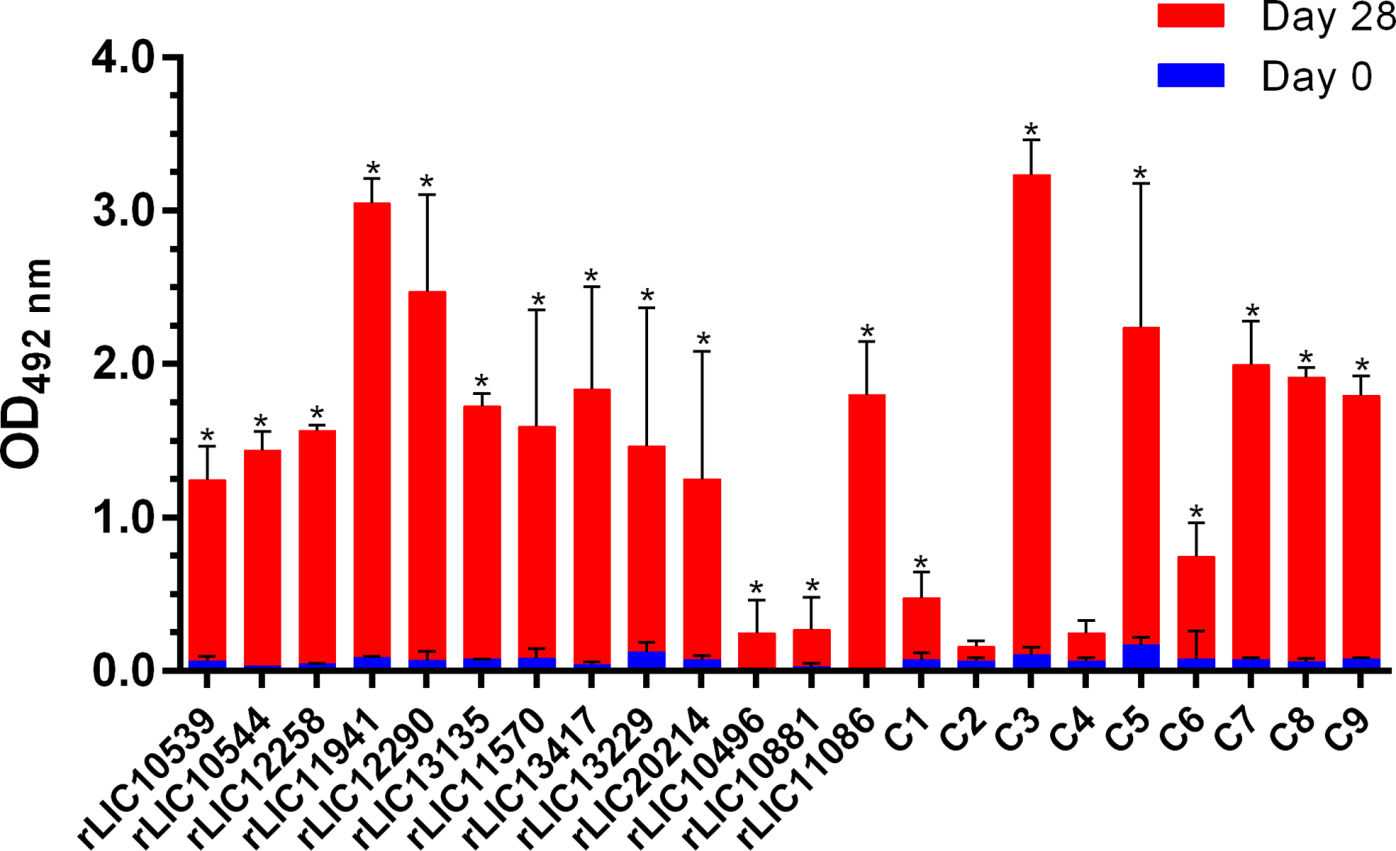
Specific humoral immune response (IgG) in vaccinated hamsters. ELISAs were carried out to determine the antibody levels in hamsters vaccinated with the recombinant proteins or the PBS control (**Table 2**). Pre-immune and pre-challenge serum samples were characterized using an anti-hamster IgG secondary antibody. The serum samples were diluted at 1:100 except for rLIC10881 and C6 (1:50), rLIC11570, rLIC13229, rLIC13417*, rLIC20214 (1:200) and LIC10496 and LIC11086 (1:400). The results represent the mean optical density (OD_492_) ± standard deviation (bars) calculated from individual serum samples assayed in triplicate. Significance differences (*P* < 0.05) were determined by one-way ANOVA (Tukey multiple comparison). *, indicates a significant difference between pre-immune sera and day 28 post immunization.

The specific humoral immune response was evaluated by ELISA using serum samples collected on day 0 (pre-immune) and day 28 post-immunization and an anti-hamster IgG secondary antibody (**Figure 4**). Of the recombinant constructs, 20/22 induced significant (*P* < 0.05) levels of IgG antibodies in vaccinated hamsters. Only the chimeras C2 (LIC10714 + LIC10881* + LIC20151) and C4 (LIC11623 + LIC12254 + LIC11268) failed to induce significant levels of circulating anti-βb-OMPs antibodies compared to the pre-immune sera (**Figure 4**). An analysis of IgG subclasses was performed on serum samples collected from animals that survived challenge (**Figure S5**). Compared to the PBS/Alhydrogel control group, immunization with rLIC11570, stimulated significant levels of IgG1 and IgG2 (*P* < 0.05). rLIC13229, and rLIC13417* induced a predominantly IgG2 response (*P* < 0.05), while rLIC20214 induced significant production of IgG2 and IgG3 (*P* < 0.05) compared to the control group. No detectable levels of antibodies were found in the pre-immune samples (data not shown).

## Discussion

The development of a universal vaccine against leptospirosis is a challenge due to the wide antigenic diversity of pathogenic *Leptospira* spp. (Picardeau, 2017; Vincent et al., 2019). RSV are *in silico*, high-throughput, approaches that identify all proteins, in a given genome, that are localised to the OM and that are surface-exposed (Rappuoli et al., 2016). RSV has been applied to a wide range of microorganisms and has achieved critical success in the discovery of novel vaccine candidates, reviewed in (Dormitzer et al., 2012). We applied this approach to the *L. interrogans* Fiocruz L1-130 genome, initially identifying 165 βb-OMPs that were refined to 18 surface-exposed, highly conserved βb-OMPs among the pathogenic *Leptospira* spp. (Grassmann et al., 2017a). For this study we focused on the βb-OMPs, often regarded as low-hanging-fruit, as they are among the easiest transmembrane proteins to predict in Gram-negative bacteria due to their 3D structure and that they are only present in the OM (Schulz, 2002). We updated the original βb-OMP list and selected 33 novel surface-exposed proteins for evaluation in the hamster model of leptospirosis. To further validate our *in silico* findings, we cross-checked them with the latest data from our CSIP experiment and found experimental evidence that six of the proteins were recognised by patient sera (**Table 1**). Immunoinformatics analysis and 3D structural modelling of the βb-OMPs allowed us to design 22 recombinant constructs such that each βb-OMP was evaluated in one or more challenge experiments. The recombinant constructs were based on either: full-length proteins; surface-exposed regions; or chimeras containing surface-related immunogenic epitopes (SRIEs) from 2 – 5 βb-OMPs. When considering a universal vaccine, one of the main criteria is that vaccine candidates should be well conserved among the pathogenic species of the target microorganism. Of the vaccine candidates selected in this study, 31/33 contained orthologues in the pathogenic *Leptospira* spp. most often associated with human disease (Vincent et al., 2019), and with AAIs > 60%, (**Figure S2** in **Supplementary Material**). Although LIC10496 was only present in *L. interrogans*, *L. kirschneri* and *L. noguchii*, we included this vaccine candidate as these three species are most likely to cause severe human leptospirosis.

In two experiments, we observed 100% immunoprotection and sterilizing immunity in hamsters vaccinated with either rLIC13229, rLIC13417, rLIC11570 or rLIC20214, see (**Figure 3A** and **Table 2**). In addition, rLIC13135, C1 (LIC10896* + LIC10964 + LIC12374) and C3 (LIC11458 + LIC11506 + LIC11086 + LIC20019) significantly increased survival in vaccinated animals compared to the control groups. Moreover, four rβb-OMPs elicited significant levels of IgG antibodies, characterized by several IgG subclasses, indicative of both Th1 and Th2 profiles (Mosmann & Coffman, 1989). These observations are in broad agreement with previous studies on recombinant subunit vaccines against leptospirosis (Lin et al., 2016; Conrad et al., 2017; Fernandes et al., 2017; Oliveira et al., 2018). However, the protective results were not reproducible in subsequent experiments, see (**Table 2**). There is little information as to the function of these proteins in *Leptospira* spp. and most are annotated as conserved hypothetical proteins. LIC13229 was reported to be excreted in the urine of infected hamsters (Segawa et al., 2014) and its gene expression was downregulated by temperature shift from 37°C to 28°C (Qin et al., 2006), suggesting that LIC13229 is involved in infection. LIC13417* gene expression was significantly downregulated in DMCs, with a possible role in the early stage of infection (Caimano et al., 2014). In agreement with our RSV analysis, several reports identified LIC20214 as an OMP and potential vaccine candidate by genome comparative and reverse vaccinology studies (Louvel et al., 2006; Viratyosin et al., 2008; Zeng et al., 2017). Over the course of 13 independent experiments, we evaluated different lots of recombinant proteins, alhydrogel adjuvant and batches of hamsters, without the success seen in the initial experiments. These findings and our previous experience together with those reported by other research groups has led us to question the suitability of the hamster model for the evaluation of vaccine candidates against leptospirosis, we discuss this further below.

OMPs are considered important cellular structures of the OM of Gram-negative bacteria and therefore represent good vaccine candidates (Silhavy et al., 2010; Sperandeo et al., 2019; Walker and Black, 2021). They are frequent targets for antibody-based therapies and vaccines for several reasons: OMPs contain surface-exposed epitopes, making them potentially accessible to antibodies or T-cell receptors; they are involved in essential cellular functions such as adhesion, biofilm formation, regulation of quorum sensing, and the export of toxic substances; these proteins tend to be conserved and highly expressed, thereby increasing their bioavailability (Maiti et al., 2020). RV uses a genomics-based approach to identify all potentially surface-exposed proteins (Rappuoli et al., 2016). SV takes RV a step further and identifies SRIEs based on the structural characteristics (3D models) of the OMPs (Cozzi et al., 2013). The ultimate goal is to identify and select protein targets that can elicit robust immune and memory responses, leading to specific and lasting protection (De Temmerman et al., 2011).

Antigen presentation other than linear epitopes has been largely overlooked during vaccine development, with a few exceptions (Faisal et al., 2009a; Faisal et al., 2009b). The use of subunit vaccine formulations could play a role in the failures observed, despite the promising results from the immunoinformatics analyses. In the current study, we evaluated three TBDRs in a recombinant chimera construct (C1). While C1 failed to induce significant protection, it did significantly increase survival (*P* < 0.05, Log-rank) compared to the control group (**Figure 3J**, **Table 2**). This can be an indication that the TBDRs were promising vaccine candidates and if they were presented to the immune system using an alternative approach, it could result in an improvement. As seen when a *Mycobacterium bovis* BCG vector vaccine expressing a chimera of these TBDRs was used to vaccinate hamsters, 100% protection and sterilizing immunity was observed (Bettin et al., 2022).

All 33 of the βb-OMPs in the current study contained putative antigenic B and T-cell epitopes on the surface-exposed regions used for construction of the chimeras. These SRIEs were predicted to induce humoral as well as cellular immunity. Despite the observation that 20/22 of the recombinant constructs were immunogenic, the use of linear epitopes may have limited their impact. The practical challenges associated with the insoluble expression of recombinant βb-OMPs resulted in their exclusion from the *Neisseria meningitidis* serogroup B vaccine (Pizza et al., 2000). However, an OM vesicle vaccine preserved the conformational epitopes of the native proteins and improved protection in clinical trials (Awanye et al., 2019). The use of OM vesicles in vaccines for the delivery of βb-OMPs is an interesting approach for the maintenance of conformational epitopes (van de Waterbeemd et al., 2010). The importance of conformational epitopes was demonstrated using a Chlamydial OM complex (COMC) from *Chlamydia muridarum* that contained several OMPs (Yu et al., 2020). The COMC vaccine was highly immunogenic and protected against infection. However, when the COMC vaccine was denatured protection was significantly reduced, suggesting conformational epitopes were required for protection. Furthermore, the first report of protection against leptospirosis reported synergy between OmpL1 and LipL41 when expressed as *E. coli* membrane-associated proteins (Haake et al., 1999).

Another potential problem is the accessibility of antibodies to βb-OMPs in Gram-negative bacteria, with several reports of a shielding effect by lipopolysaccharides (LPS), resulting in the lack of a protective immune response (van der Ley et al., 1986; Bentley and Klebba, 1988; Murphy et al., 1990; Michaelsen et al., 2001; Patel et al., 2016; Dominguez-Medina et al., 2020). This shielding effect was proposed as an evolutionary advantage for Gram-negative pathogens. Patel and colleagues modelled the ability of LPS to interact with the *E. coli* OmpF polypeptide, effectively camouflaging its epitopes from host immune recognition during infection (Patel et al., 2016). Antibody accessibility to OM proteins in *Leptospira* spp. is another issue that should be considered, especially given its unusual LPS composition (Vinh et al., 1986; Que-Gewirth et al., 2004).

Antibodies have several biological effects against extracellular pathogens, such as neutralization, phagocytosis, antibody-dependent cellular cytotoxicity and complement-mediated lysis (Heesterbeek et al., 2018; Siegrist, 2018). In the current study, the induction of significant levels of total IgG were observed for 20/22 of the recombinant constructs evaluated (**Figure 4**). However, the immunoglobulin levels did not correlate with survival, as previously observed with other vaccine candidates (Coutinho et al., 2011; Monaris et al., 2015; Conrad et al., 2017; Raja et al., 2018). In contrast to the bacterin vaccines, the available data for recombinant vaccines suggests that a humoral response is not sufficient to clear the bacteria from the host. These findings suggest that a more complex immune response is required to control the infection, perhaps one involving cellular immunity. Furthermore, positive correlations were observed between TNF-α, IL-10, IL-4, IL-12p40 and IFN-γ mRNA levels and heterologous protection in animals vaccinated with RecA and FliD using a prime-boost protocol; a RecA/FliD recombinant vaccine only induced partial protection (Raja et al., 2018). Again, there was no observable association between antibody levels and protection. While it is only possible infer cytokine levels in the hamster model by quantitative real-time RT-PCR, the potential lack of correlation between mRNA levels and protein abundance means that data interpretation requires caution (Koussounadis et al., 2015; Liu et al., 2016).

Recombinant proteins produced in *E. coli* are often contaminated with LPS, this endotoxin is a known stimulator of the immune system and is the main cause of septic shock during a bacterial infection. The presence of endotoxins in vaccine preparations is strictly controlled during clinical trials as even trace amounts can have a major effect (Petsch and Anspach, 2000; Wakelin et al., 2006). Given the ability of endotoxins to stimulate the immune system, their presence in recombinant vaccines requires further study. Of note, the C3H/HeJ mouse model is resistant to LPS shock, further supporting its use as an alternative model for the evaluation of vaccine candidates (Gomes-Solecki et al., 2017; Shetty et al., 2021; Kundu et al., 2022).

The absence of correlation between antibody levels and protection induced by recombinant vaccines, as well as a potential role for cellular immunity in a protective immune response, represent key challenges for the discovery of vaccine candidates. The lack of immune correlates is a major limitation to screening the hundreds of novel targets identified by RSV and CSIP (Grassmann et al., 2017b; Felix et al., 2020; Vernel-Pauillac et al., 2021). It is only possible to screen significantly reduced numbers of vaccine candidates using animal models, with the possibility that promising targets may be missed. The discovery of an immune correlate would substantially reduce the use of animals and allow the screening of hundreds of targets. However, despite the evaluation of opsonophagocytosis assays developed in other spirochaetes such as *Treponema* and *Borrelia* spp. (Cruz et al., 2008; Hawley et al., 2017), this remains elusive for *Leptospira* spp.

Syrian hamsters (*Mesocricetus auratus*) have long been used as a model to isolate *Leptospira* spp., recover virulence in laboratory-attenuated strains, investigate aspects of pathogenesis, screen for the virulence of mutant strains and to evaluate vaccine candidates in research laboratories (Haake, 2006; Zuerner, 2015). Additionally, hamsters are used to evaluate the efficacy of commercial vaccines against leptospirosis (Srinivas et al., 2013). They are the preferred model as hamsters are susceptible to the disease, recapitulate the symptoms of severe human leptospirosis and are easily bred in animal units, while mice and rats are naturally resistant to leptospirosis, reviewed in (Gomes-Solecki et al., 2017). Nevertheless, the absence of immune correlates and the lack of commercially available reagents to study the immune response in hamsters has hampered further advances in the field (Adler, 2015b; Felix et al., 2020). Hamsters are notoriously susceptible to leptospirosis and can succumb to infection with as little as one leptospire (Haake, 2006). The hamster model is therefore of limited use as a model of sublethal or chronic infection.

In contrast, most natural hosts of leptospires develop chronic infection with kidney colonization, urinary shedding and little or no clinical signs of disease (Athanazio et al., 2008; Richer et al., 2015; Zuerner, 2015; Gomes-Solecki et al., 2017). The mouse model has been used for the evaluation of vaccine candidates; it was used in the first evaluation of a LigA vaccine (Koizumi and Watanabe, 2004). Importantly, there is an abundance of commercially available reagents for the characterisation of the immune response. In addition, there are mouse mutants e.g., the C3H/HeJ TLR4 mutant, SCID and *Rag1* KO mice, which are susceptible to leptospiral infection (Bandeira et al., 2011; Shetty et al., 2021; Grassmann et al., 2021; Kundu et al., 2022). The use of a mouse model would allow the study of both lethal and sublethal forms of leptospirosis, potentially allowing the discovery of immune correlates that can be used to screen vaccine candidates (Pereira et al., 1998; Santos et al., 2010; Gomes-Solecki et al., 2017; Felix et al., 2020).

The limitations of the current study include the following: the influence of endotoxins on the immune response induced by the recombinant vaccine preparations remains unknown and requires further study; due to a lack of commercially available reagents, the cellular response cannot be evaluated in the hamster model; and the lack of immune correlates for leptospirosis is a major limitation for the screening of vaccine candidates *in vitro*. The use of the C3H/HeJ mouse model could resolve the first two limitations; it is susceptible to leptospirosis, does not recognise *E. coli* LPS, and there are a wide range of commercially available reagents available to study the humoral and cellular immune response. Furthermore, these reagents could assist in the development of an immune correlate for leptospirosis.

## Conclusion

We report the use of RSV and CSIP for the identification of vaccine candidates in the *L. interrogans* Fiocruz L1-130 genome and the evaluation of 22 vaccine candidates (based on 33 βb-OMPs) against leptospirosis, four of which induced significant protection in two independent experiments. Furthermore, these recombinant proteins stimulated significant humoral immune responses and sterilizing immunity in immunized hamsters. In addition, two chimera constructs significantly increased survival in vaccinated animals, suggesting they have potential as vaccine candidates. However, when we tested the reproducibility of these vaccine candidates in additional experiments, they failed to induce protective immune responses. These results, together with other reports in the literature have led us to question the suitability of the hamster model of leptospirosis for the evaluation of vaccine candidates. We further propose that alternatives such as the mouse acute and chronic models should be re-evaluated.

## Material and methods

### Bacterial strains and cultivation

*E. coli* strains were grown in liquid Luria-Bertani (LB) medium (180 rpm) or on solid LB medium at 37°C. Ampicillin (100 µg/ml) and chloramphenicol (34 µg/ml) were used for selection when necessary. *L. interrogans* serogroup Icterohaemorrhagiae serovar Copenhageni strain Fiocruz L1-130 was maintained at 28°C in liquid Ellinghausen-McCullough-Johnson-Harris (EMJH) (Difco, BD, Brazil) supplemented with Leptospira enrichment EMJH commercial supplement (Difco, BD, Brazil).

### Functional annotation and sequence conservation among orthologs

The list of 165 putative βb-OMPs previously identified by our research group RSV was updated as described (Grassmann et al., 2017a). Functional annotation was performed using UniProt and InterProScan (Zdobnov and Apweiler, 2001). Orthologues were identified in genome sequences from 20 additional *Leptospira* spp., using the reciprocal best hit (RBH) method based on protein BLAST (BLASTp) searches (Altschul et al., 1997) as previously described (Grassmann et al., 2017a). Protein sequences with >70% similarity and >40% coverage were considered orthologous. A multiple sequence alignment was performed with the orthologues from the available pathogenic *Leptospira* spp. using the Clustal Omega tool (Sievers et al., 2011). When this analysis was carried out there were 10 pathogenic *Leptospira* spp. genomes available, see (**Figure S2** in **Supplementary Material**), corresponding to node 1 (9 species) in the P1 subclade of pathogenic *Leptospira* spp. based on the most recent genome diversity study and *L. alsontii* (Vincent et al., 2019).

### Structural modelling and functional annotation

Given the likely importance of phagocytosis and clearance of leptospires during the infection, we confirmed the presence of SRIEs in the βb-OMPs. This was dependent on the ability to predict the orientation of the βb-OMPs in the OM, and this was achieved by analysing the closest PDB models with the orientation of proteins in membranes database. These epitopes are therefore likely to be exposed on the leptospiral surface and be capable of binding to MHC class II receptors, thereby stimulating the host immune response. The closest structural analogues in PDB of each 3D model generated by I-TASSER were used as references to determine the orientation of the leptospiral βb-OMPs in the OM, as well the probable surface exposed regions. The presence of MHCII epitopes (HLA-DRB alleles) and linear B cell epitopes in the fragments exposed on the OM was predicted using NetMHCII software and BepiPred-2.0, respectively (Lundegaard et al., 2008).

### βb-OMP structural membrane allocation and epitope predictions

To identify proteins containing β-barrel transmembrane domains, conserved sequences among pathogenic species were subjected to 3D modelling by protein threading using I-TASSER server (Zhang, 2008). I-TASSER results from the top-ranking models with barrel structures were used to identify the PDB entries with the closest structures to the target protein models. For protein 3D models with secondary or tertiary I-TASSER rankings, a 3D structure-based functional annotation was performed using COFACTOR (Roy et al., 2012), as previously described (Grassmann et al., 2017a). The 3D structures were visualized using UCSF Chimera software V. 1.11.2 (Pettersen et al., 2004).

### Identification of vaccine candidates by cell-surface immunoprecipitation (CSIP)

This technique was used to experimentally identify leptospiral proteins localized on the cell surface of host-adapted *L. interrogans*, as described previously (Cunha et al., 2017). Briefly, we produced host-adapted leptospires (*L. interrogans* Fiocruz L1-130) by cultivating them within dialysis membrane chambers that were surgically implanted in the peritoneal cavity of Wistar rats and cultivated for 9 – 12 days (Caimano et al., 2014; Grassmann et al., 2015). Intact, host-adapted leptospires were recovered and surface-exposed proteins were immunoprecipitated using pooled human sera from convalescent leptospirosis patients. Serum samples were kindly donated by the Public Health Central Laboratories (LACEN) in Rio de Janeiro (RJ) and Porto Alegre (RS) in Brazil. Leptospirosis was confirmed by the microscopic agglutination test (MAT) and ELISA (WHO and ILS, 2003). Following recovery, the immunoprecipitated proteins were identified using mass spectrometry (Newcombe et al., 2014).

### Design and cloning of the β-barrel OMPs

The predicted 3D models of the βb-OMPs were used to design the recombinant proteins in this study. We used three strategies to clone the vaccine candidates: 1) Cell-surface exposed regions of 10 βb-OMPs (without transmembrane or periplasmic regions) for the following proteins: LIC10539, LIC10544, LIC11570, LIC11941, LIC12258, LIC12990, LIC13135, LIC13229, LIC13417* and LIC20214; 2) Three full-length proteins: LIC10496, LIC10881* and LIC11086; and 3) Cell-surface exposed regions from 29 proteins in nine multi-chimeric constructions as follows: chimera 1 (C1): LIC10896* + LIC10964 + LIC12374; chimera 2 (C2): LIC10714 + LIC10881* + LIC20151; chimera 3 (C3): LIC11458 + LIC11506 + LIC11086 + LIC20019; chimera 4 (C4): LIC11623 + LIC12254 + LIC11268; chimera 5 (C5): LIC10496 + LIC12575 + LIC11211 + LIC13417*; chimera 6 (C6): LIC10496 + LIC10881*; chimera 7 (C7): LIC10539 + LIC10544 + LIC12258; chimera 8 (C8): LIC11271 + LIC11366 + LIC11975 + LIC12252; chimera 9 (C9): LIC11941 + LIC12290 + LIC13135 + LIC12693 + LIC12307. The chimera constructs were designed to include a range of protein functions in each construct. For design strategies 1 and 3, the surface-related regions were synthesised in series without linkers, see (**Table S2** in **Supplementary Material**). The regions from each protein were assembled using Vector NTI v.11 and commercially produced, including codon optimization for *E. coli* expression, and cloned into the expression vector pAE (Ramos et al., 2004), using *Bam*HI, *Kpn*I or *Hin*dIII restriction sites.

### Recombinant protein expression and purification

Engineered recombinant proteins were expressed in *E. coli* BL21 (DE3) Star or pLysS cells as described previously (Bettin et al., 2022). Briefly, the product of each *E. coli* heat shock transformation was cultivated in LB medium containing 100 μg/ml of ampicillin and chloramphenicol (34 µg/ml for *E. coli* pLysS), at 37°C. When cultures reached mid-log phase (OD_600_ 0.6 – 0.8), protein expression was induced with the addition of IPTG for 3-4 h. Pellets were suspended in lysis buffer (0.2 M NaH_2_PO_4_, 0.5 M NaCl and 20 mM imidazole, pH 8.0), sonicated by 6× 30s cycles on ice and centrifuged (11.000 x *g*, 40 min at 4°C). After lysis, soluble recombinant proteins were purified directly from the supernatant. For the recovery of insoluble proteins (contained in inclusion bodies) the pellets resulting from the post-lysis centrifugation were solubilized in a denaturing buffer (lysis buffer, 8 M urea). The recombinant proteins were purified by immobilized metal ion affinity chromatography (IMAC) using HisTrap FF columns (GE Healthcare, Brazil) using an AKTA Start chromatography system (GE Healthcare, Brazil), as described previously (Conrad et al., 2017). The fractions containing the soluble proteins were pooled and dialyzed against PBS pH 7.5, or 50 mM Tris pH 8.5 buffer at 4°C for up to 24 h. The fractions containing insoluble proteins were dialyzed against PBS pH 7.5 or 50 mM Tris pH 8.5 containing 0.05% Triton X-100 at 4°C. Protein concentrations were determined using a BCA protein assay kit (Thermo Fisher Scientific, Brazil) and the proteins were stored at –20°C.

### Determining the challenge dose for the hamster model

Male and female Syrian hamsters (*Mesocricetus auratus*) were used as the animal model for acute leptospirosis. The challenge dose for the pathogenic species *L. interrogans* serogroup Icterohaemorrhagiae serovar Copenhageni strain Fiocruz L1-130 was determined using 8-week-old hamsters, as described previously (Silva et al., 2007). Briefly, groups of three hamsters were infected by intraperitoneal injection with 10^0^–10^3^ leptospires in 1 ml EMJH medium. Leptospires were quantified using a Petroff-Hauser counting chamber and darkfield microscopy and only motile leptospires were counted. Hamsters were monitored daily for clinical signs of leptospirosis over a period of 28 days. Endpoint criteria included: 10% weight loss (see **Figure S4**), nasal bleeding, prostration and failure to respond to stimulation (Coutinho et al., 2011). Animals that fulfilled one or more of the endpoint criteria were immediately euthanized by CO_2_ narcosis. The endpoint dose that caused endpoint criteria in 50% of infected animals (ED_50_) was calculated as previously described (Reed and Muench, 1938).

### Evaluation of immunoprotection in hamster model of lethal leptospirosis

The vaccine preparations for the cell-surface exposed regions and the full-length proteins contained 50 µg recombinant protein, while the chimeric constructs contained 25 µg of each protein in the construct (**Table 2**); this was based on previously published data, see e.g., (Silva et al., 2007; Conrad et al., 2017). For vaccine formulations, recombinant proteins were prepared to a 15% (v/v) final concentration of aluminium hydroxide adjuvant (2% Alhydrogel, *In vivo*Gen, USA) and gently mixed for 16 h at 4°C. For immunizations, male and female Golden Syrian hamsters aged 4-6 weeks, were randomly allocated into groups of 9-10 animals each. Animals were immunized by intramuscular injection with two doses at 14-day intervals. To evaluate vaccine-induced protection, hamsters were challenged intraperitoneally 28 days after the first immunization with 10 – 20× ED_50_ of *L. interrogans* Fiocruz L1-130. The animals were monitored 3× daily for up to 28 days PC. Animals that developed endpoint criteria or that survived to day 28 PC were euthanized by CO_2_ narcosis. Pre-immune blood samples were collected on day 0, prior to vaccination, and day 28, prior to challenge, and stored at –20°C. Thirteen independent experiments were performed to evaluate the 22 recombinant protein constructs.

### Leptospiral renal burden

Kidney samples were collected, macerated and inoculated into EMJH medium as previously described (WHO and ILS, 2003). Cultures were periodically examined by dark-field microscopy for up to twelve weeks before being considered negative. The leptospiral renal burden was evaluated by quantitative real-time PCR (qPCR) as previously described (Conrad et al., 2017), with the following differences. Genomic DNA was extracted from the kidneys of infected animals using the SV Genomic DNA Purification kit (Promega, Brazil). DNA from *L. interrogans* Fiocruz L1-130 was extracted using the Illustra Bacterium Genomic Prep Mini Spin kit (GE Healthcare, Brazil) and quantified using Quant-iT dsDNA Assay Kit and the Qubit fluorometer (Thermo Fisher Scientific Inc., USA), following the manufacturer’s instructions. The purified genomic DNA was diluted to generate a standard curve ranging from 2×10^1^ to 2×10^6^ copies/reaction. Reactions were performed in triplicate using LipL32-f 5’-CTGAGCGAGGACACAATC and LipL32-r 5’-ATTACGGCAGGAATCCAA primers.

### Evaluation of the humoral immune response

The induction of the antibody-based immune response was evaluated by indirect ELISA using purified recombinant proteins as previously described (Conrad et al., 2017), with the following modifications: polystyrene 96 well microtitration plates were coated with 50-200 ng/well of each individual recombinant protein. The plates were blocked with 5% non-fat milk solution in PBS-T, and hamster sera were added at 1:50 – 1:400 dilution. Peroxidase-conjugated anti-Syrian hamster IgG antibody (Jackson ImmunoResearch, USA) or anti-IgG subclasses (anti-IgG1, IgG2/3 and IgG3) (Southern Biotech, USA) were used as the secondary antibodies. Reactions were developed by adding o-phenylenediamine dihydrochloride (Sigma-Aldrich, Brazil) and hydrogen peroxide and stopped with addition of 3N H_2_SO_4_. Optical density was read at 492 nm and mean values were obtained from serum samples assayed in triplicate.

### Statistical analysis

Protection against lethal leptospirosis and survival rates were evaluated using the two-tailed Fisher’s exact test (GraphPad QuickCalcs) and the Log-rank test (GraphPad Prism), respectively. Antibody levels were analysed with ANOVA to compare differences between the groups (Tukey’s multiple comparisons). GraphPad Prism v.8. was used to perform statistical analysis, and *P* values < 0.05 were considered significant.

## Data availability statement

The original contributions presented in the study are included in the article/**Supplementary Material**. Further inquiries can be directed to the corresponding author.

## Ethics statement

All animal experimentation was conducted following the Brazilian Guide for the Production, Maintenance and Use of Animals for Teaching Activities and Scientific Research, adhering to international guidelines. All protocols were reviewed and approved by the ethics committee on the use of animals (CEUA Nos: 8230-2017, 59050-2018 and 19193-2018) at the Federal University of Pelotas (UFPel). The CEUA at UFPel is accredited by the Brazilian National Council for Animal Experimentation Control (CONCEA). The hamsters used in the current study were provided by the animal unit at UFPel.

## Author contributions

OD, JM, and AM contributed to conception and design of the study. MM, EB, LB, and NO performed the bioinformatics analysis, carried out the statistical analyses and wrote the first draft of the manuscript. MM, EB, LB, NO, TB, AP, GR, ER, and AS performed the laboratory experiments. EB, NO, and AM created the figures and tables. All authors contributed to manuscript revision, read, and approved the submitted version.

## Funding

This work was supported by grant IC170171 from The Royal Society - UK, grants 311212/2021-2 and 315550/2020-1 from the Brazilian National Council for Scientific and Technological Development (CNPq) and grant 001 from the Coordenação de Aperfeiçoamento de Pessoal de Nível Superior - Brazil (CAPES).

## Conflict of interest

The authors declare that the research was conducted in the absence of any commercial or financial relationships that could be construed as a potential conflict of interest.

## Publisher’s note

All claims expressed in this article are solely those of the authors and do not necessarily represent those of their affiliated organizations, or those of the publisher, the editors and the reviewers. Any product that may be evaluated in this article, or claim that may be made by its manufacturer, is not guaranteed or endorsed by the publisher.
