# Supplementary material for: Challenges for the development of a universal vaccine against leptospirosis revealed by the evaluation of 22 vaccine candidates
Source: Front Cell Infect Microbiol. 2022 Oct 7;12:940966. doi: 10.3389/fcimb.2022.940966 (PMC9586249; doi:10.3389/fcimb.2022.940966)
Supplement: Supplementary file 2 [file DataSheet_1.docx]

***Supplementary Material***

The evaluation of 22 vaccine candidates highlights the challenges that need to be overcome in the discovery of a universal vaccine against leptospirosis

Mara A C Maia, Everton B Bettin, Liana N Barbosa, Natasha R de Oliveira, Tiffany T Bunde, Ana Carolina K Pedra, Guilherme A Rosa, Elias E B da Rosa, Amilton C P Seixas Neto, André A Grassmann, Johnjoe McFadden, Odir A Dellagostin, Alan J A McBride*

*** Correspondence:** Dr. Alan McBride, alan.mcbride@ufpel.edu.br

# Supplementary Data

Supplementary data is distributed in three Figures and two Tables.

# Supplementary Figures and Tables

## Supplementary Tables

**Supplementary Table S1**. [EXCEL FILE] Epitope prediction for the selected leptospiral beta-barrel-OMPs using NetMHCII and BepiPred servers.

**Supplementary Table S2**. [WORD FILE] Sequences of the selected regions in the βb-OMPs predicted to contain surface-exposed regions, and B and T cell epitopes. Protein sequences were submitted to BepiPred and NetMHCII for prediction of linear B cell (bold) and T CD4+ cell (underlined) epitopes, respectively. All surface-exposed loops containing identified epitopes were selected to compose the fusion protein and are listed. A methionine amino acid followed by a polyhistidine-tag (6xHis-tag-MHHHHHH) and additional four amino acids (LEGS) (from the pAE vector multiple cloning site), were auditioned before each recombinant construction and are highlighted in red.

**Supplementary Table S2.** Epitope prediction for the selected leptospiral beta-barrel-OMPs using NetMHCII and BepiPred servers.

| **Protein** | **Selected Fragments Sequence** | **Amino Acids localization in the native proteins*** | **Number of *in tandem* repeats in recombinant construct**** | **Predicted molecular mass for the recombinant proteins (kDa)** |
| --- | --- | --- | --- | --- |
| LIC10496 (Full-length) | Recombinant sequence:  *MHHHHHHLEGS*TTKQPPIKITLDQAVLIGSSNSVVLKVLEAKK**EVSKMLITEKWREFLPKFGIQYYGLRNQNVNSADNIYNDI**RLTVQQLIFD**G**G**EANL**NLEIAK**L**SE**LLNEQDFKINL**SRLRLDIQKAYFRALALKGKVYIQ**KKAQEKAQEALRKGQVELRQGFITKVQLMD**LESKL**KQTEFNVQKSKNDSDQ**ALLDLKQVM**NLDYYAEIELNESIFFDFIINAPPNTHNLDE**LISKAKNGR**EDLKKMQIIVKKLKNEKEVLDNQYM**PKVYVGAYA**GRNGNNNQFTHDSYGVNFNLVMPLGSSVVQSNGNTGVQKDGNGIQTYPGFGNQTVGPGT**NSYNSTSVRL**FDNLSQSRKAMEGEIQLAEALLNYRN**MENQVGFEIK**K**S**VDKLNQSWELINIANSRINLQVES**GRAMAAKV**AYGHAKKEDQI**NSELEMIK**SQEDLTD**ALTSYAINCYEYAQIT**TDEGGLRKLIQYSKGSGNSILSNLIKNQETGSKP**KK | 27-509 | - | 55.82 |
| LIC10539 | Recombinant sequence:  *MHHHHHHLEGS*GNGTEQSELRPFPYKTPNSGSTTRLRQTLAFGTSSESESSGERTVNAILGEMNTKFQKRMWTRTSQEVDSIGQKTGSGIDPEWKFLKRFSLQGNQFGLDYRWKSIGTEIFMETSNMNLKNTLQTGKILIPMLYESKTILTYVQV |  |  | 17.67 |
|  | Surface-exposed fragments:  GNGT**EQSELRPF** | 141-152 | - |  |
|  | **PYKTPNSGSTTRL**R | 175-188 |  |  |
|  | QTLA**FGTSSESESSGERTV**NAILG**EMNTKFQK** | 213-244 |  |  |
|  | **RMWTRTSQEVDSIGQKTGSGIDP** | 269-291 |  |  |
|  | **EWKFLKRFSLQG** | 313-324 |  |  |
|  | **NQFGLDYRWKSIGTEI** | 354-369 |  |  |
|  | **FMETSNMNLKNTLQT**G | 384-399 |  |  |
|  | KILIPMLYESKTILTYVQV | 419-437 |  |  |
| LIC10544 | Recombinant sequence:  *MHHHHHHLEGS*HNPYRSSSRDNRYLTQPARWDEVNSNYKNEVSSEKNSNEVSVRNLALDYVPYYSNGWSNITETNKDKAVGNEAPDSQARQTRYQRVSDPGVQAYRQWYMFDPEQTIIQTASKNGFLTSFRSYDAVFDRDQSKSKEEK |  |  | 17.34 |
|  | Surface-exposed fragments:  HNP**YRSSSRDNRYLTQPARWDE** | 133-154 | - |  |
|  | **VNSNYKNEVSSEKNSNEVSV**RN | 183-204 |  |  |
|  | LALDYV**PYYS** | 246-255 |  |  |
|  | **NGWSNITETNKDKA** | 276-289 |  |  |
|  | **VGNEAPDSQARQTRY** | 310-324 |  |  |
|  | **QRVSDPGVQAYRQWY** | 349-363 |  |  |
|  | **MFDPE**QTIIQ**TASKNGFLTS** | 386-405 |  |  |
|  | **FRSYDAVFDRDQSKSKE**EK | 428-446 |  |  |
| LIC10881*  (Full-Length) | Recombinant sequence:  *MHHHHHHLEGS*GTLWAKASLRLKIFANSKSDPPTQIWVRGNGYSKV**FSLSD**QSSELTIDL**K**E**Q**GVYDVILTFKSGEMEHKFV**TVDSDEKNLEFVQKTKKVTN**GINVVG**KRPEAPPNYVLSQEDAIRMPGGFGDALKAVQSMPGISPMYQMYTGASFQSAIQTFAQATNPDKPDKPNGEK**GFLVMRGAGARANQFYFNGLPMSY**PFHADGLTSV**IN**NN**AIRSLELYS**GSYSARYGF**ATGGIINIEGF**QKRDSNL**SVAHLNAFL**TDVYTYRNITKDLN**VSVSGKK**YYPNIVFGRVPNLIPAETFLADYN**DYQARIGW**DISEN**HSLSFQTFGA**KDKRYPFKELSQYNPKETAQSFANPPSDADAARLDRIFRTDGIQHIWKPKSS**ITNTFN**V**S**RNYFNEVTENGLDMLVLDITKIGYPPSLYKRVQTIQNEYFNDLRQIENVSEVELLKRN**WKIVFGG**QYREVDTGYKGKVSQIDLDPTYNFIHQQLLNSSDVKSVLEGDSV**RTRQIGTFFENR**FKFY**DFNLNLGVRR**EYYDK**SRE | 36-557 | - | 60.56 |
| LIC11086  (Full-Length) | Recombinant sequence:  *MHHHHHHLEGS*HHTGMGGSDQSSTRFVDPFTGKREKPANYVVLTQDFFKQTNENSNIHTSTFFGEINLKNGMFALNLSVPYTYYEQKDRSDAARIGKTYIGIKYLPLVDFQKNYFIVFSANVGFPSGPDTDKFTGGNYYSGIPGLTFGYLLGKFSFVGKLSGIFPLSKSQPSNLQDNDGIVYWLRNPSSSPPEETYLLKKTSLFSGYVTYLWKPGLSFFTGFLYRTPYEGVDLKRSNQGKVPSIFREISLGFSANISEKLNFNLSYRYPLYRGEDYRLYDYAITAAVSIEISELENSKPAKVEEVKQEPEETTQETK | 23-328 | - | 36 |
| LIC11570 | Recombinant sequence:  *MHHHHHHLEGS*RYKGEAFGNSNGQVNPNIAGSEQIIRYKGEAFGNSNGQVNPNIAGSEQIIRYKGEAFGNSNGQVNPNIAGSEQIIRYKGEAFGNSNGQVNPNIAGSEQIIRYKGEAFGNSNGQVNPNIAGSEQIIRYKGEAFGNSNGQVNPNIAGSEQIIRYKGEAFGNSNGQVNPNIAGSEQIIRYKGEAFGNSNGQVNPNIAGSEQII |  | 8x | 22.52 |
|  | Surface-exposed fragments:  RYKG**EAFG** | 338-345 |  |  |
|  | **NSNGQVNPNI** | 359-368 |  |  |
|  | **AGSE**QII | 381-387 |  |  |
| LIC11941 | Recombinant sequence:  *MHHHHHHLEGS*EPTQRYFDFSRSRGGTAFQNYWGEPTQRYFDFSRSRGGTAFQNYWGEPTQRYFDFSRSRGGTAFQNYWGEPTQRYFDFSRSRGGTAFQNYWGEPTQRYFDFSRSRGGTAFQNYWGEPTQRYFDFSRSRGGTAFQNYWGEPTQRYFDFSRSRGGTAFQNYWGEPTQRYFDFSRSRGGTAFQNYWGEPTQRYFDFSRSRGGTAFQNYWG |  |  | 26.13 |
|  | Surface-exposed fragments:  EPTQR**YFDFSRS** | 123-134 | 9x |  |
|  | **RGGTAFQ**NYWG | 335-345 |  |  |
| LIC12258 | Recombinant sequence:  *MHHHHHHLEGS*TTFFNGIDYTFWKAWKWVVANAGSIWETPVPNGESKLTEDYRAGKIRGYNGGDIVYFVSKRTGSDFNYNKYTSSGAPFSEVRYMWSLDGPMTGIGGLQTMRGYRQDRFVAPVVGVGRVWDSEKRINLQGYKHSFAKSKEDSQMF |  |  | 17.75 |
|  | Surface-exposed fragments:  TTFFN**GIDY**TFWKAWKWVVAN | 184-204 | - |  |
|  | AG**SIWETPVPNGESKLTEDYRAGKI**RGYNGGDIV**YF** | 227-262 |  |  |
|  | **VSKRTGSDFNYNK** | 290-302 |  |  |
|  | **YTSSGAP**FSEVRYMWSL**DGPMTGIGGLQTMRGYRQ**DRFVAPVVG | 331-374 |  |  |
|  | VGRV**WDSEKRINLQGYKHS** | 404-422 |  |  |
|  | **FAKSKEDSQ**MF | 441-451 |  |  |
| LIC12290 | Recombinant sequence:  *MHHHHHHLEGS*RSAANESGSEEDRSGVANRRSAANESGSEEDRSGVANRRSAANESGSEEDRSGVANRRSAANESGSEEDRSGVANRRSAANESGSEEDRSGVANRRSAANESGSEEDRSGVANRRSAANESGSEEDRSGVANRRSAANESGSEEDRSGVANRRSAANESGSEEDRSGVANRRSAANESGSEEDRSGVANRRSAANESGSEEDRSGVANRRSAANESGSEEDRSGVANR |  |  | 25.04 |
|  | Surface-exposed fragments:  RSAA**NESGSE** | 109-118 | 12X |  |
|  | **EDRSGV**ANR | 307-315 |  |  |
| LIC13135 | Recombinant sequence:  *MHHHHHHLEGS*RIPGPNSHFNPFYTEPNPLTGSSSTTSSLPPTVEHNTTPNKRIPGPNSHFNPFYTEPNPLTGSSSTTSSLPPTVEHNTTPNKRIPGPNSHFNPFYTEPNPLTGSSSTTSSLPPTVEHNTTPNKRIPGPNSHFNPFYTEPNPLTGSSSTTSSLPPTVEHNTTPNKRIPGPNSHFNPFYTEPNPLTGSSSTTSSLPPTVEHNTTPNKRIPGPNSHFNPFYTEPNPLTGSSSTTSSLPPTVEHNTTPNK |  |  | 27.78 |
|  | Surface-exposed fragments:  RIPGPN**SHFNPFYTEPNPLTGSSSTTSSLPPTV** | 303-335 | 6X |  |
|  | **EHNTT**PNK | 528-535 |  |  |
| LIC13229 | Recombinant sequence:  *MHHHHHHLEGS*EAKYNYDFDKFKNDNISFVTIWGGEKGSPNGLDTANDNTRQFVMIVGEQDSDVAGNSTNLGKKNSFPIQYNCPPNSTSFSTCTFSAELSKQQQGDSTFTIGLYKKWFPQNNSTILLLPNPETIPRDSRYDQLHTFRQTGRNGINITPSWDTFNTNISTVDPLTGKTITNNVYKERQAYDAYALGSGDPNRKDGKVTTFSNLFHSNHGFFGEADQVSWVNMIGIVDKHKRQDGWYDVTGALKEGASTESITNERFKDPYVQTEKGALDQRSVVTLGKNLFRESWIFAGDAVRRKLNDDSISPEFRKTQFLPQAQFG |  |  | 36.80 |
|  | Surface-exposed fragments:  EAKYN**YDFDKFKN**DNISFV | 92-110 | - |  |
|  | TIW**GGEKGSP**NGLDTANDNTRQFV | 136-159 |  |  |
|  | MIVG**EQDSDVAGNSTNLGKKNSFPIQ**YNCPP**NSTSFS**TCTFSAE**LSKQQQGDSTFT** | 223-278 |  |  |
|  | I**GLYKKWFPQN**NSTILLLPNP**ETIPRDSRY**DQLHTF | 297-332 |  |  |
|  | R**QTGRNGINITPSWDTFNTNISTVDPLTGKTITNNVYKERQAYD**AYA | 358-404 |  |  |
|  | L**GSGDPNRKDGKVTTFSNLFHS**NHGFFGEADQVSWV**NMIG** | 424-463 |  |  |
|  | **IVDKHKRQDGWYDVTGALKEGASTESITNERFKDPYVQTEKGALDQRSVV**TLGKNLFRE | 484-542 |  |  |
|  | SWIFAGD**AVRRKLNDDSISPEFRKTQFLPQA**QFG | 561-594 |  |  |
| LIC13417* | Recombinant sequence:  *MHHHHHHLEGS*SGALLSQTAQVTENKPSVESPAATKQVTDTKPEDADYVSPMKKEGVSAEYNRYESRNNLNFDKSNHAYTDRIVWGGASPANVGDIRSGFFSREAFVMIKKLPLDAKVQVGRARVMFDYDSFSSHFFVARPFWTQSGVNGVVSANDPKLNSTVANTNPSASQSDTDVVKKWIPQTTPSSTADDVLAQNRKKQNDMLYTTGFRLTNRTNKNNLPQGRGWDGYTFAEKLRIGAQYTYASGDNNRADGSISTFQTLVNPRFGVIYKNEKWGMFQIAYIINDKVQKNDAWAGSTENYTGNSFTQPYSTGRNIYNAGNSIRNYRNSPLIYNTTTNEFELNSAAFMKQHTSAN |  |  | 39.90 |
|  | Surface-exposed fragments:  SGALL**SQTAQVTENKPSVESPAATKQVTDTKPEDADYVSPMKKEGVSAEYNR** | 24-75 | - |  |
|  | **YESRNNLNFDKSNHAYT**DRI | 110-129 |  |  |
|  | VWGG**ASPANVGDIRSGF**FS | 156-174 |  |  |
|  | REAFVMIK**KLPL**DAKVQVGR | 201-220 |  |  |
|  | ARVMFDYDSFSSHFFVARPFW**TQSGVNGVVSANDPKLNSTVANTNPSASQSDT** | 246-298 |  |  |
|  | **DVVKKWIPQTTPSSTADDVLAQNRKKQND**MLYTTGF**RLTNRTNKNNLPQGRGWD** | 321-374 |  |  |
|  | **G**YTFAEKLRIGAQYTY**ASGDNNRADGSISTFQTLVNP**RF**GVI** | 429-470 |  |  |
|  | **YKNEKW**GMFQIAYIIND**KVQKNDAW** | 497-521 |  |  |
|  | **AGSTENYTGNSFTQPYST**GRNIYN | 553-576 |  |  |
|  | AG**NSIRNYRNSPLIYNTTTNEFELN**SAA**FMKQH**TSAN | 601-637 |  |  |
| LIC20214 | Recombinant sequence:  *MHHHHHHLEGS*VPVAAFGGGANNYSFRGADPNTNLYPFHFDGLTGYIDKTFETLGTLAGGSLLPEGVRLPRYTDAKDDFALDPPAKRQNDPTKDELAGFAGGNLSAGQGYRSYDPFVDFNVSFGSIKAKQRGSGARFLNYKTTGSTIQQTDPNNPDPNPYDTTSPDFKTVPDTNRVQSKYYDYIPYIKNGAGAGNHFNFPLDTRFSEQSGNPHLKFQKAFQEFSNLIVTDPYITEAIGTNPDQYGRITQPYILNKPLSYSNNGTGHSRSQTFRNPNIYKPDAIMTQVATGSEQRLVAQTFPNSKETLYDYDRTHMTSTPITPIVGDDGGKFSNPANNQTIWIPVSGNNPYLAEYTNSKRLKDYHYMRENVSGENFDVTRPYSATNPKPNPTFGTLTLPGGAIIPF |  |  | 44.84 |
|  | Surface-exposed fragments:  VPVAAFG**GGANNYSFRGADPNT**N | 154-176 | - |  |
|  | LYPFHFDGLT | 186-195 |  |  |
|  | GY**IDKTFETLG**TLAGGSLLPEG**VRLPRYTD** | 269-298 |  |  |
|  | **AKDDFALDPPAKRQNDPTKDE**LAGFAGG**NLSAGQGYR** | 320-356 |  |  |
|  | **SYDP**FVDFNVSFGSIK**AKQRGSG**A | 378-401 |  |  |
|  | RFLNY**KTTGSTIQQTDPNNPDPNPYDTTSPDFKTVPDTNRVQSKY**Y | 426-471 |  |  |
|  | DYIPYI**KNGA** | 493-502 |  |  |
|  | **GAGNHFNFPLDTRFSEQSGNPHLKFQKAF** | 526-554 |  |  |
|  | **QEF**SNLIVTDPY**ITEAIGTNPDQ**YGRITQPYILN**KPLSYSNNGTGHSR** | 577-624 |  |  |
|  | **SQTFRNPNIYKPDAIMTQVATGSEQ**RLVAQTF**PNSKETLYDYDRTH** | 650-695 |  |  |
|  | **MTST**PITPIVGDDGGKFSNPANNQTIWIPVS**GNNPYLAEYTNSKRLKDYH** | 718-767 |  |  |
|  | **YMRENVSGENFDVTRPYSATNPKPNPT**FGTLTLPGGAIIPF | 794-834 |  |  |
| **Chimera C1** | Recombinant sequence:  *MHHHHHHLEGS*YNRSYNYREEANARFQASNPISIYLKDSNMLRPLNQNNLKIYGEEVLWGNNLNLAYEPKIGQQFFIKTLYSVQSDKIVREGDGANYIDNFNFKSTNLNFIWSQGGTDLANGYRRLGNNPDGTRREIAQRNFTGSDRDVIYPIPGEVIYNPLAYANGNRKIYERSWNGFNTSYGCKTNSEEERLLLVRANICDATDSKIYGLISTGQVDPLSTYAAYSPTTLNRPLQGQSDDLLDNPNENPGATVSQKLLHEKSHFHSFFIFSAGNLELDFSYQRIQNFIYAASIAQIDLDSGLPKYEYKQGNDQNFSYKNDHGTVVLNTLDDTIDRRKNASFPSEEPWYRRQDPLSGDIK  Surface-exposed fragments:  LIC10896:  YNRS**YNYREEANARFQAS**NPISIYLKDSNM**LRPLNQNNLKIYGEEVLWGNNLNLAYEPKIG**QQFFIKTLYSVQ**SDKIVREGDGANYIDNFNFKST**NLNFI  W**SQGGTDLANGYRRLGNNPDGTR**  **REIAQRNFTGSDR**DVIYPIPGEVIYNPLA**YANGNRKIYER**  **SWNGFNTSYGCKTNSEE**ERLLLVRANICDAT  DSKIYGLIST**GQVDPLSTYAAYSPTTLNRPLQG**QSD  LIC10964:  DLLD**NPNENPGATVSQKLLHEKSH**FHSFFIFSAG**NLELDFSYQ**R  IQNFIYAASIAQIDLD**SGLPKYEYKQGN**  LIC12374:  DQNF**SYKNDHGTVVLNTLDDTIDRRKNAS**  **FPSEEPWYRRQDPLSGDIK** | 360-459  505-527  573-612  663-693  849-884  330-373  597-624  210-238  421-439 | 1x  1x  1x | 41.34 |
| **Chimera C2** | Recombinant sequence:  *MHHHHHHLEGS*PPTPDYAIFGNGFNNPPTGVASSEYSLAKSGNVRTILRQQTIDPVTNQPDPNSPSQEVDKGSKRADARMNQYSIDTSKITADQSLLDFVVTQKDKNGNYVPYVSKDTRNYFNEVTENGLDMLVLDITKIGYPPSLYKRVQTQIDLDPTYNFIHQQLLNSSDVKSVLEGDSVRTPYAMDPFSRNRDLMRESLDPNADLSLVRRSNTPVVGSKSITSEQFNSQLLPSAAPPPPSSSSNSSSSLFSIYQPVYSPGYRLVPNSQATTGNDTRDLSSDRPLEGRALHSSTNNLSAAGQDYIPSEVKLNENPPVIYDNLLNQYELAYNPTRPRFYY  Surface-exposed fragments:  LIC10714:  PPTPDYAIFGN**GFNNPPTGVASSEYSLAKSGN**V  RTILR**QQTIDPVTNQPDPNSPSQEVDKGSK**  **RADARMNQYSIDTSKITAD**QSLLDFVV**TQKDKNGNYVP**YVSKDT  LIC10881:  RNYFNEVTENGLDMLVLDITK**IGYPPSLY**KRVQT  **QIDLD**PTYNFIHQQLLNSSDVK**SVLEGDSVRT**  **PYAMDPFSR**NRDLMRE**SLDPNA**DLSLVRRSN  **TPVVGSKSITSEQF**NSQL**LPSAAPPPPSSS**SNSSSSLFSIYQPVYS  LIC20151:  PGYR**LVPNSQATTGN**  **DTRDLSSDRPLEGRALH**  **SSTNNLSAAGQDYIPSEVKLNE**NPPVIY  DNLLNQY**ELAYNPTRPR**FYY | 416-448  488-517  658-701  415-448  494-525  642-672  803-848  255-269  584-600  630-657  684-703 | 1x  1x  1x | 37.97 |
| **Chimera C3** | Recombinant sequence:  *MHHHHHHLEGS*VDRGALYSADPNAPLRNIGPDVEPWSYEYLRTSSNLRFGIPLLFFNTTYRKTEIYKPELQDPILGKTRQHRWTPYDYTSYTERSILETTIKTDSIKQNQKVLIPEIELANPNTNVIESGTLGYETRKQYFNPMLRSTQGTRFYKPQALALESITVSAGTAGAQGIYNYIYSYFSYYSFHQTDIRLQTSSLDPFISENSSAPILHMFRSGNHDILQSKSQPSNLQDNDGIVYWLRNPSSSPPEETYLLKKTFETGTKYPNLSGLKGGFFLGDPTVERGTKISTREFSYYDTPYTFIGSRNFADGKG  Surface-exposed fragments:  LIC11458:  VDRGA**LYSADPNAPLRNIGPDVEPW**  **SYEYLRTSSN**LRFGIPLLFFNT**TYRKTEIYKPELQDPILGKT**RQH  LIC11506:  RWTP**YDYTSYTERSILETTIKTDSIKQNQKV**LIP  E**IELANPNTNVIESGTLGYETRKQYFNPMLRS**  **TQGTRFYKPQALALESITVS**AG**TAGAQGIY**  **NYI**YSYFSYYS**FHQTDIRLQTSSLDPFISENSSA**PILH**MFRSGNHD**ILQ  LIC11086:  **SKSQPSNLQDN**DGIVYWLR**NPSSSPPEETY**LLKKT  LIC20019:  FETG**TKYPNLSGL**KGG  FFLGD**PTVERGTKISTREFSYYD**TPYTFIGS**RNFA**DGKG | 380-404  641-685  58-91  111-142  217-246  270-318  178-212  87-102  148-186 | 1x  1x  1x  1x | 36.14 |
| **Chimera C4** | Recombinant sequence:  *MHHHHHHLEGS*IYNVGAVSITENNNQQSIKEQAIYSRIQKPYIQLQDLQFLGGYESLRGWFYNDAKYPAEWRDGAALYEEVNRATGVRKDLFETYDQRVREAQMKDPVGYYLANNYNLTALRKADYTFEETANNSVSIKNNTDLREGFVQLKNFADGLNNPANLVLSGNNVALDKFAQKLKYTGVADHPFTKFESDNAFQFNGKTKVTEDAEAGKIIGANGGNVKSAFGLTEGDAPFFEYRNLWSTEGGITGLGGLRTLRGYKQDRFDYSHNADFDKRTQDDRNYATQNSQ  Surface-exposed fragments:  LIC11623:  IYNVGAVS**ITENNNQQSIKEQA**IYSR  IQKPYIQLQDLQ**FLGGYESL**RGW**FYNDAKYPAEWRDGAA**  LYEEVNRATGVRK**DLFETYDQRVREAQMKDPV**GYYL**ANNYNLTALRKADYTFEELNNP**ANLVLSGNN**VALD**K  FA**QKLKYTGVADHPFTKFESDNA**FQF  LIC12254:  NGKT**KVTEDAEAG**KIIGANGGNVKS  AF**GLTEGDAPFF**EYRN**LWSTEGGITGLGGLRTLRGY**KQDR  LIC11268:  FDYS**HNADFDKRTQDDRNYATQNSQ**  **TANNSVSIKNNTDLRE**GFVQLKNFADG | 607-632  781-819  848-919  941-966  301-325  391-431  120-144  196-222 | 1x  1x  1x | 33.01 |
| **Chimera C5** | Recombinant sequence:  *MHHHHHHLEGS*NNNQFTHDSYGVNFNLVMPLGSSVVQSNGNTGVQKDGNGIQTYPGFGNQTVGPGTILPFNQANFFTGTKISDDTIKGGIEKVLQTTGTYFKIEAGSRRFDSNAFENPSTTPAGFSSLGIPPFGQETEEQSKFYGPYLKVSTGFPSFDSQLEKKLNTYETPKRDYIGDSYTAEDRKTKINGLEYSTSEANELIANGRTLDVITIKSIDKTTNVRGAVIALDSTGRPGFDKDGNRANLKGGGYKTLMPGYSISNIANDFTGGYALFSGKDSSG  Surface-exposed fragments:  LIC10496:  NNNQ**FTHDSYG**VNFNLVMPLGS**SVVQSNGNTGVQKDGNGIQTYPGFGNQTVG**PGT  LIC12575:  ILPFNQANFFT**GTKISDDTIKGG**I**EKVLQT**TGTYFK**IEAGSRRFDSNAFENPSTTPAGFSSL**GIPP  LIC11211:  FGQE**TEEQSKFYG**PYLKVSTG**FPSFDSQLEKKLNTYE**  **TPKRDYIGDSYTAEDRKTKINGLEYSTSEANELIANGRT**LDVI  LIC13477:  TIKSI**DKTTN**VRGAVIALD**S**  **TGRPGFDKDGNRANLKGGGYKTLMPGYSISNIANDFTG**GYALF**SGK**DSSG | 297-351  93-158  129-165  234-276  269-288  370-419 | 1x  1x  1x  1x | 30.48 |
| **Chimera C6** | Recombinant sequence:*MHHHHHHLEGS*RNQNVNSADNIYNRNGNNNQFTHDSYGVNFNLVMPLGSSVVQSNGNTGVQKDGNGIQTYPGFGNQTVGPGTGFGDALKISPMYQMYTGASFQSAIQTFAQATNPDKPDKPNGEKGFLVMRGAGARANQFGFATGLKKYYPNIVFGRVPNLIPAETFLADYNDYQARFGAKDKRYPFKELSQYNPKETAQSFANPPSDADAARLDRIFRTVSRNYFNEVTENGLDMLVLDITKIGYPPSLYKRVQTIQNEYFNDREVDTGYKGKVSQIDLDPTYNFIHQQLLNSSDVKSVLEGDSVRTRQIEYYDKSRE  Surface-exposed fragments:  LIC10496:  RNQN**VNSADNIYN**  **RNGNNNQFTHD**SYGVNFNLVMPLG**SSVVQSNGNTGVQKDGNGIQTYPGFGNQTVGPGT**  LIC10881:  **GFGD**ALK  ISPMYQMYTGASFQSAIQTFAQAT**NPDKPDKPNGE**KGFLVMRGAGARANQF  GFAT**G**  L  **KK**YYPNIVFGRVPNLIPAETF**LADYNDYQAR**  **FGAKDKRYPFKELSQYNPKETAQSFANPPSDADAA**RLDRIFRT  VSRNY**FNEVTEN**GLDMLVLDITKI**GYPPSLYKRVQTIQNEYFND**  REVDTGYKGKVSQIDLDPTYNFIHQQLLNSSDVKSVLEGDSVRTRQI  **EYYD**KSRE | 58-70  268-325  154-160  168-218  258-262  286  306-336  352-394  413-456  482-528  550-557 | 1x  1x | 35.83 |
| **Chimera C7** | Recombinant sequence:  *MHHHHHHLEGS*TTFFNGIDYTFWKAWKWVVANAGSIWETPVPNGESKLTEDYRAGKIRGYNGGDIVYFVSKRTGSDFNYNKYTSSGAPFSEVRYMWSLDGPMTGIGGLQTMRGYRQDRFVAPVVGVGRVWDSEKRINLQGYKHSFAKSKEDSQMFHNPYRSSSRDNRYLTQPARWDEVNSNYKNEVSSEKNSNEVSVRNLALDYVPYYSNGWSNITETNKDKAVGNEAPDSQARQTRYQRVSDPGVQAYRQWYMFDPEQTIIQTASKNGFLTSFRSYDAVFDRDQSKSKEEKGNGTEQSELRPFPYKTPNSGSTTRLRQTLAFGTSSESESSGERTVNAILGEMNTKFQKRMWTRTSQEVDSIGQKTGSGIDPEWKFLKRFSLQGNQFGLDYRWKSIGTEIFMETSNMNLKNTLQTGKILIPMLYESKTILTYVQV  Surface-exposed fragments:  LIC12258:  TTFFN**GIDY**TFWKAWKWVVAN  AG**SIWETPVPNGESKLTEDYRAGKI**RGYNGGDIV**YF**  **VSKRTGSDFNYNK**  **YTSSGAP**FSEVRYMWSL**DGPMTGIGGLQTMRGYRQ**DRFVAPVVG  VGRV**WDSEKRINLQGYKHS**  **FAKSKEDSQ**MF  LIC10544:  HNP**YRSSSRDNRYLTQPARWDE**  **VNSNYKNEVSSEKNSNEVSV**RN  LALDYV**PYYS**  **NGWSNITETNKDKA**  **VGNEAPDSQARQTRY**  **QRVSDPGVQAYRQWY**  **MFDPE**QTIIQ**TASKNGFLTS**  **FRSYDAVFDRDQSKSKE**EK  LIC10539:  GNGT**EQSELRPF**  **PYKTPNSGSTTRL**R  QTLA**FGTSSESESSGERTV**NAILG**EMNTKFQK**  **RMWTRTSQEVDSIGQKTGSGIDP**  **EWKFLKRFSLQG**  **NQFGLDYRWKSIGTEI**  **FMETSNMNLKNTLQT**G  KILIPMLYESKTILTYVQV | 184-204  227-262  290-302  331-374  404-422  441-451  133-154  183-204  246-255  276-289  310-324  349-363  386-405  428-446  141-152  175-188  213-244  269-291  313-324  354-369  384-399  419-437 | 1x  1x  1x | 50.06 |
| **Chimera C8** | Recombinant sequence:  *MHHHHHHLEGS*QSSKIKTHSVAGIGYKGEDRLNSFLRGFTNTYDPGVASKTELDPPKQTTQYREWQKFGLKQFSSEPFYTDLKFKISNPYGMSQVYWDSEGYATSGKETFRQEGLLSGTGIEFRFSWINIPSLSGTFNGESASFYLRENGSITPLTESDNQTSMLVTNQFSRKLEFQVLTTTQIECSGAACTVIPSNISSQFNGLEYEIHTKYLNEVVDFQVGAGLSAAGVKNDDGWLTGNGPADQKDKDEKDDGGRSFLHRINEMNLGYSPTQIPKISFGPATGRWDADFALDYRSGNSNIHLGVTGPMVLTLDSHGDAKAKDSGVSSGPGFNVTDRMYNRYTLSNNIVHVNDGKFVKSVDPIFEGTPLSNSGQVPNAKTKLTEDAEAGKILGYHGGFVNTKASKTIASDFDFQKISDGNVPFFEYRNLWGTENTVSGLGGLRTLRGYKQDRFVGRAMRVWNDEHKAGLTDYKYAVSREDKQLFGASNPLPGTETQKYLDTTLGAFYANYLSATERALTTIPVYGGEAFVVARPSNTSRWDPMMIVDYNKIYESRMDAPGETKYPYTSPYEDSRLSNPNYYRVVDTEFFQF  Surface-exposed fragments:  LIC11366:  QSSKI**KTH**SV  A**GIGYKGEDRLNS**FL**RGFTNTYDPGVASKTELDPPKQTTQ**  **YREWQKFGLKQFSSEPFYTDLKFKISNPY**  **GMSQVYWDSEGYATSGKETFRQEGLL**SGTGIEFR  FSW**INIPSLSGTFNGES**ASFYLRE**NGSITPLTESDNQTSMLV**TNQF**SRK**LEFQVLTTT  LIC11975:  QIECSGAACTVIP**SNISSQFNGLEY**EIHTKY**LNEVVD**  **FQVGAGLSAAGVKNDD**  **GWLTGNGPADQKDKDEKDDGGR**SFLHRIN  **EMNLGYSPTQIPKISFGPATGRWDAD**FA**LDYRS**  **GNSN**IHLGVTGPMVLTLD  **SHGDAKA**KDS  LIC12252:  GVSSGPGFNVTDRMYNRYT  LSNNIVHVN**DGKFVKSVDPIFEGTPLSNSGQVPNAKTKLTEDAEAGKI**LGYH**GGFVNT**  **KASKTIASDFDFQK**  **ISDGNVPFFE**YRNLWGT**ENTVSGLGGLRTLRGYKQDRF**VGRAM  RVWNDEHKAG**LTDYK**  **YAVSREDK**QLF  LIC11271:  GASN**PLPGTETQKY**LDTTLG  AFYANYLSATERALTTIPVY  GGEAFVVARP**SNTSRW**DPMMIV  DYNKIY**ESRMDAPGETKYPYTSPYEDSRLSNPNYYRV**V**DTE**FFQF | 28-37  57-96  117-145  168-201  222-279  34-70  94-109  140-168  238-270  302-319  345-354  235-253  280-337  365-378  407-449  482-496  518-526  102-121  140-159  180-201  220-264 | 1x  1x  1x  1x | 65.96 |
| **Chimera C9** | Recombinant sequence:  *MHHHHHHLEGS*TTLPANRNNIFAGTKQSQDKLSVGIEKNFRTGTYAKLEASTTRFDTSAFENPSTTPSNLAALAIPPLNIVSPQQNLTDGNRGVASFEPTQRYFDFSRSRGGTAFQNYWGEPTQRYFDFSRSRGGTAFQNYWGEPTQRYFDFSRSRGGTAFQNYWGPGGGSSGPQILGANGSQIKGNEHPGGGSSGPQILGANGSQIKGNEHPGGGSSGPQILGANGSQIKGNEHPGGGSSGPQILGANGSQIKGNEHRIPGPNSHFNPFYTEPNPLTGSSSTTSSLPPTVEHNTTPNKRIPGPNSHFNPFYTEPNPLTGSSSTTSSLPPTVEHNTTPNKRSAANESGSEEDRSGVANRRSAANESGSEEDRSGVANRRSAANESGSEEDRSGVANRRSAANESGSEEDRSGVANR  LIC12693:  TTLPANRNNIFAG**TKQSQDKLSVGIEKNFRTGTY**AKLEAS**TTRFDTSAFENPSTTPSNLAALAIPPL**  **NIVSPQQNLTDGNRGV**ASF  LIC11941:  EPTQR**YFDFSRS**  **RGGTAFQ**NYWG  LIC12307:  PGGG**SSGPQILGANGSQ**  **IKG**NEH  LIC13135:  RIPGPN**SHFNPFYTEPNPLTGSSSTTSSLPPTV**  **EHNTT**PNK  LIC12290:  RSAA**NESGSE**  **EDRSGV**ANR | 108-174  377-395  123-134  335-345  135-151  350-355  303-335  528-535  109-118  307-315 | 1x  3x  4x  2x  4x | 44.06 |

## Supplementary Figures S1-S5


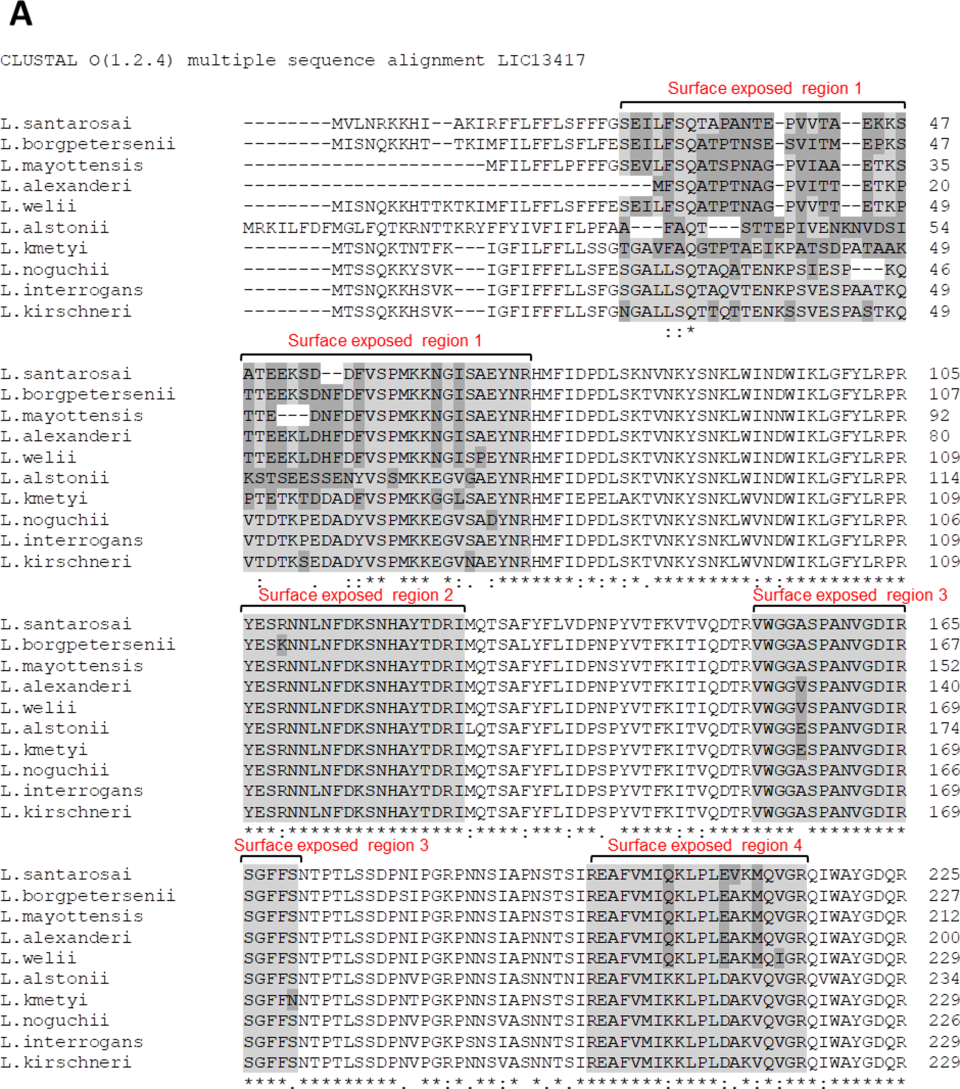


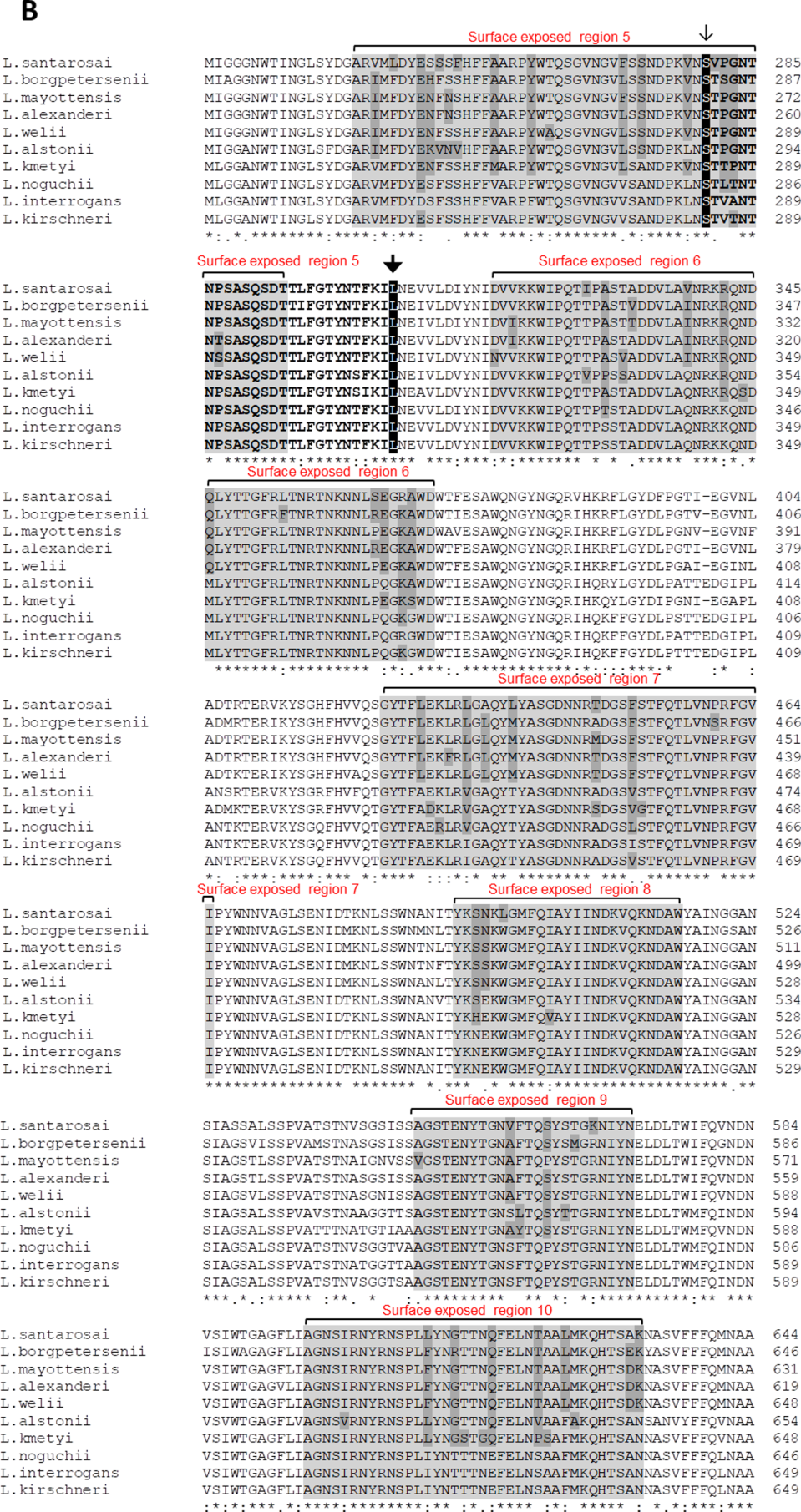


**Figure S1A-B.** LIC13417 multiple sequence alignment performed by Muscle with orthologues from other pathogenic *Leptospira* spp. The selected surface-related regions are highlighted (conserved *L. interrogans* amino acids are marked in light grey, non-conserved amino acids are marked in dark grey). The serine codon included in the modified LIC13417* amino acid sequence is conserved among orthologues of this protein and is highlighted in black. The thin arrow points to the stop codon in LIC13417. The thick arrowhead indicates the initiation codon of the LIC13418 sequence. Both amino acids are marked in black. A fragment of 25 amino acid residues (indicated in bold), located between LIC13417 and LIC13418, was omitted by the gene's annotation errors and was identified with Artemis.


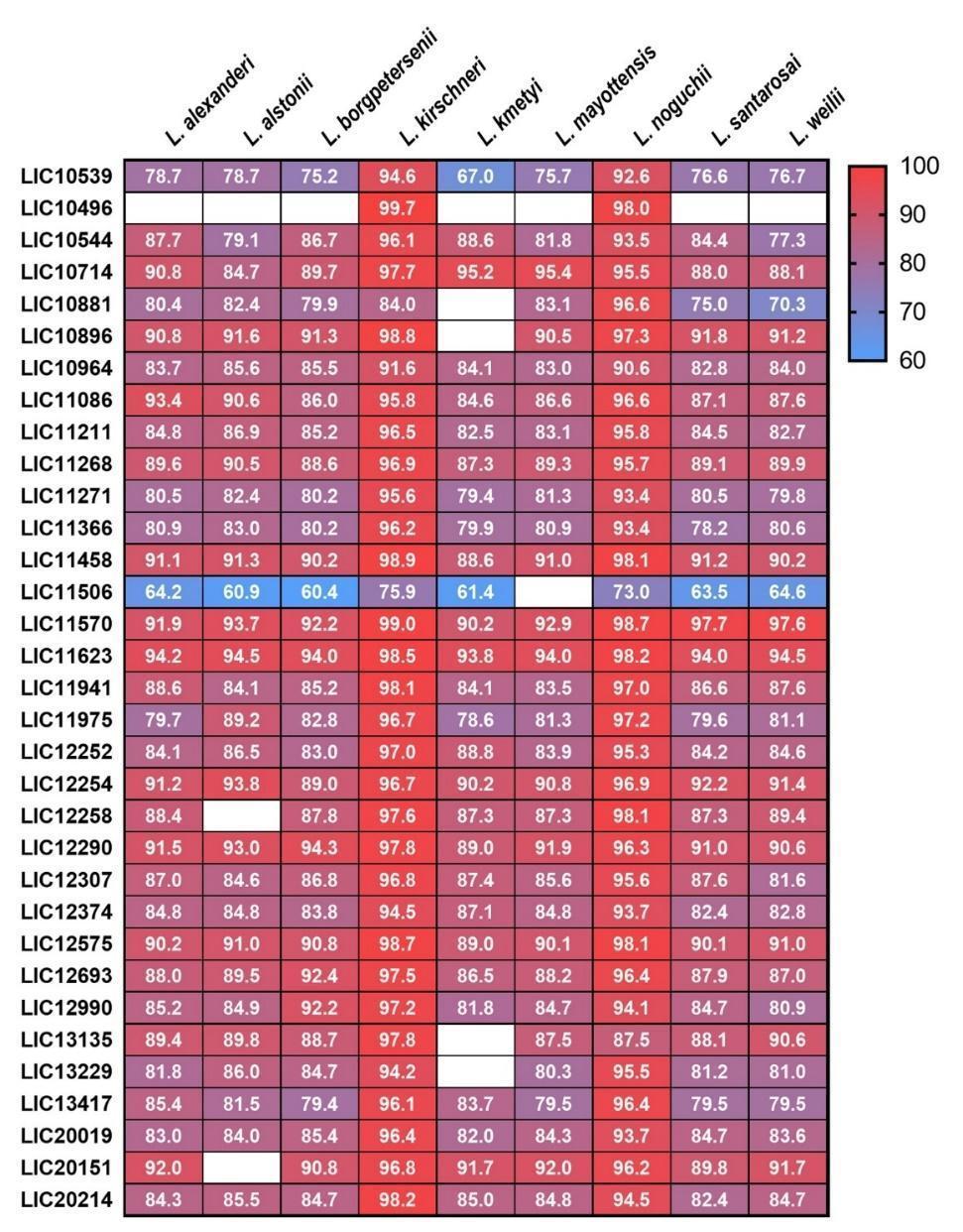


**Figure S2. Amino acid sequence conservation of the βb-OMPs among pathogenic *Leptospira* spp.** The sequences were compared using the reciprocal best hit (RBH) method based on protein BLAST (BLASTp) searches. Protein sequences with >70% similarity and >40% coverage that were also the best reciprocal hit were considered orthologues. *L. interrogans* strain Fiocruz L1-130 proteins were used as the query sequences. Blank squares signify no known orthologue.


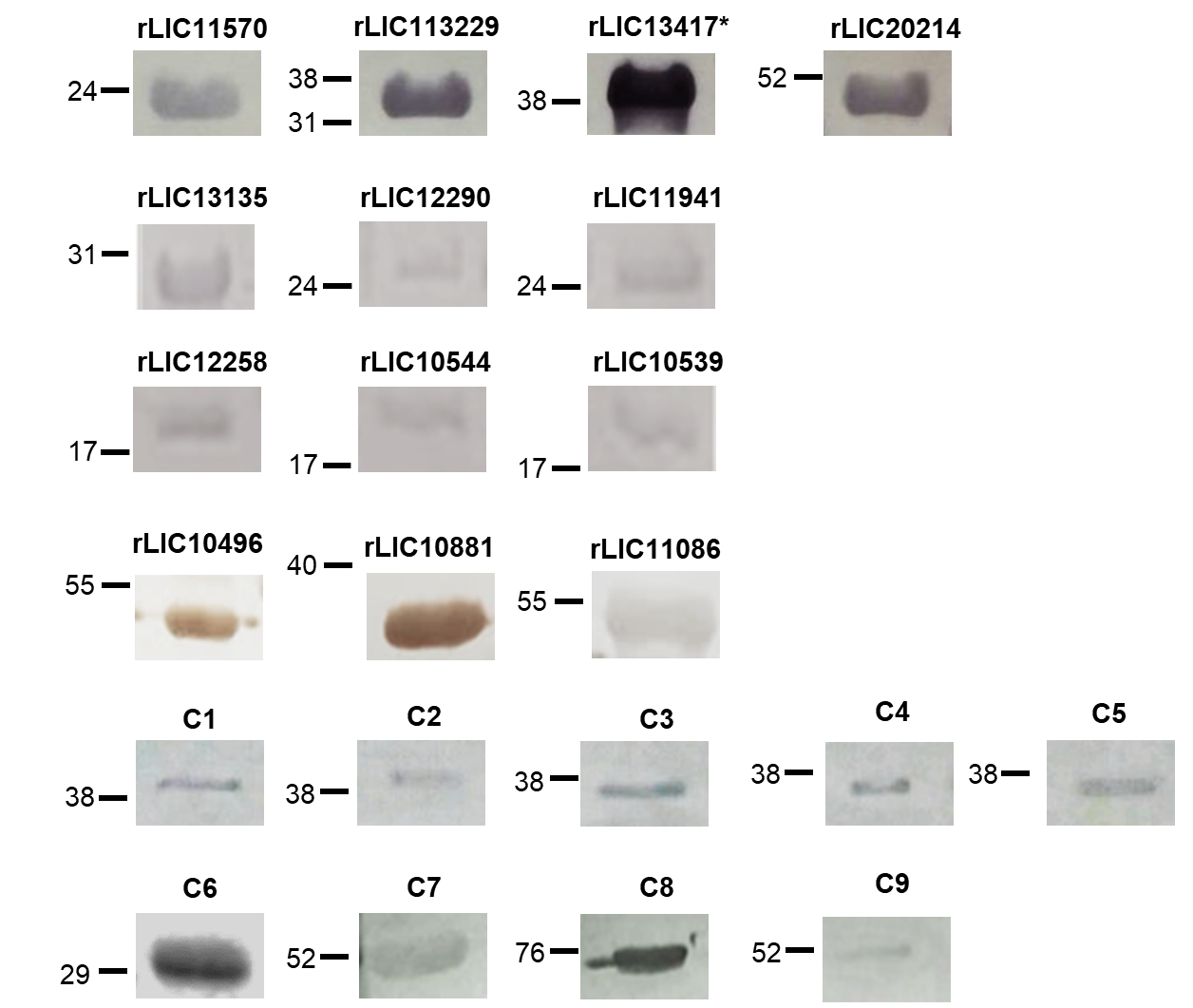


**Figure S3. Characterization of the 22 recombinant proteins evaluated in the hamster model.** Western blot analysis of the 22 recombinant constructs, following transfer the nitrocellulose membranes were probed with an HRP-conjugated 6x-His tag antibody.


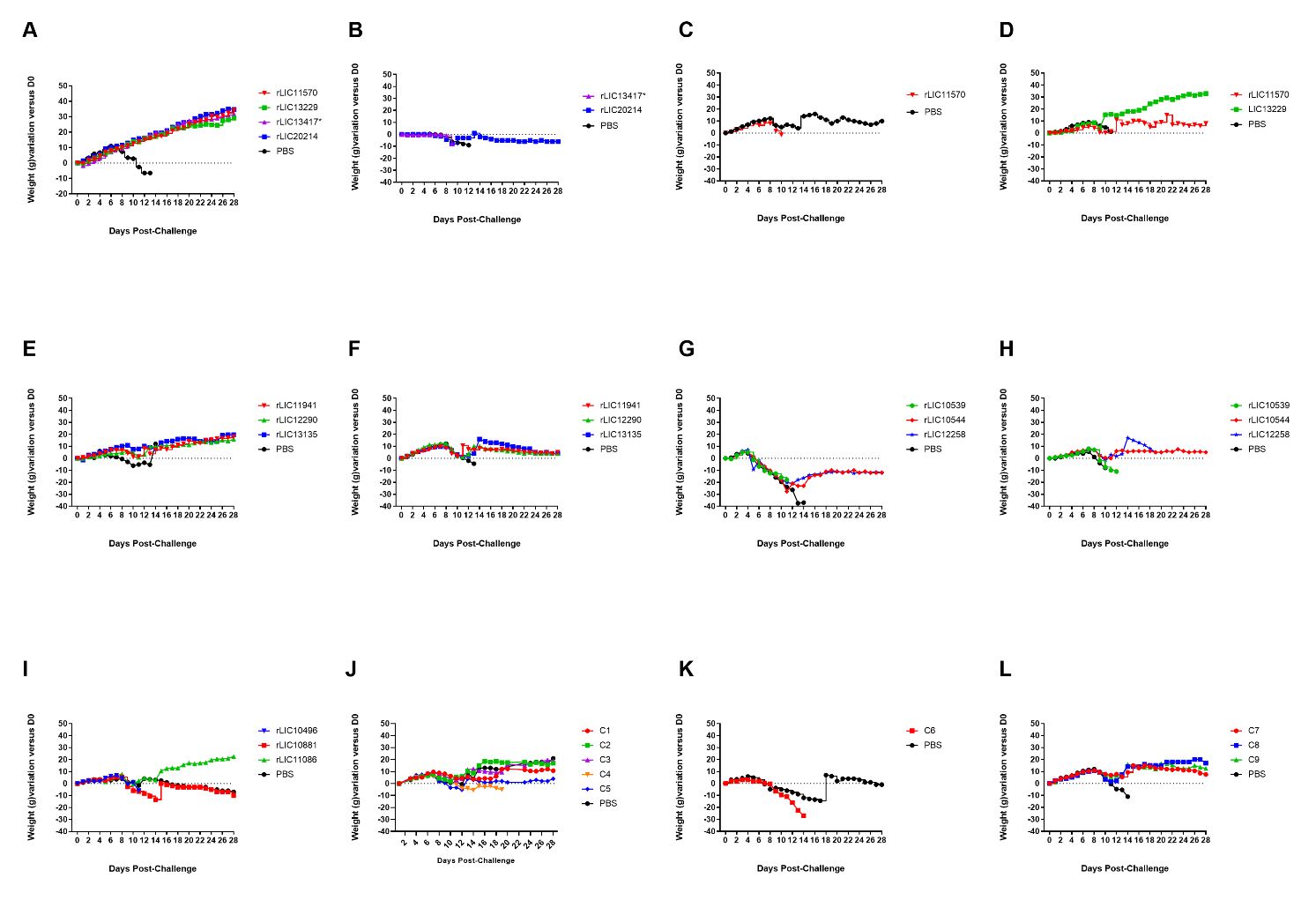


**Figure S4. Hamster weight change following challenge with *L. interrogans*.** Representative experiments showing survival of hamsters vaccinated (days -28 and -14) with two doses of the vaccine candidates or injected with a PBS/Alhydrogel control, followed by challenge (day 0) with a lethal dose of *L. interrogans* serovar Copenhageni strain Fiocruz L1-130. Weight change from the initial weight at the day 0 (D0) of infection, expressed in grams, was recorded daily for 28 days post-challenge. Groups of hamsters were vaccinated with: A) rLIC11570 (GspD), rLIC13229 (AlgE), rLIC13417* (AlgE), rLIC20214 (TBDR) or the PBS control. All the rβb-OMPs induced 100% protection (P < 0.0001) and sterilizing immunity; B-D) repeat experiments failed to reproduce the protective immune response seen previously; E) vaccinated with the OEPs rLIC11941, rLIC12290, rLIC13135 or the PBS/Alhydrogel control. While the level of protection was the same (44.4%) for all proteins, only rLIC13135 significantly increased survival (P < 0.05) among vaccinated hamsters compared to the PBS control group; F) a repeat experiment using the OEPs failed to reproduce these results; G-H) vaccinated with rLIC10539 (Omp85), rLIC10544 (porin), rLIC12258 (Omp85) or the PBS/Alhydrogel control. None of the vaccine preparations protected against the challenge dose; I, vaccinated with rLIC10496 (TolC), rLIC10881* (TBDR), rLIC11086 (AlgE) or the PBS/Alhydrogel control. None of the vaccine preparations protected against the challenge dose; J, hamsters were vaccinated with chimeras C1-C5 or the PBS/Alhydrogel control. K-L, hamsters vaccinated with chimeras C6-C9 or the PBS/Alhydrogel control group. None of the chimeras induced a significant protective immune response.


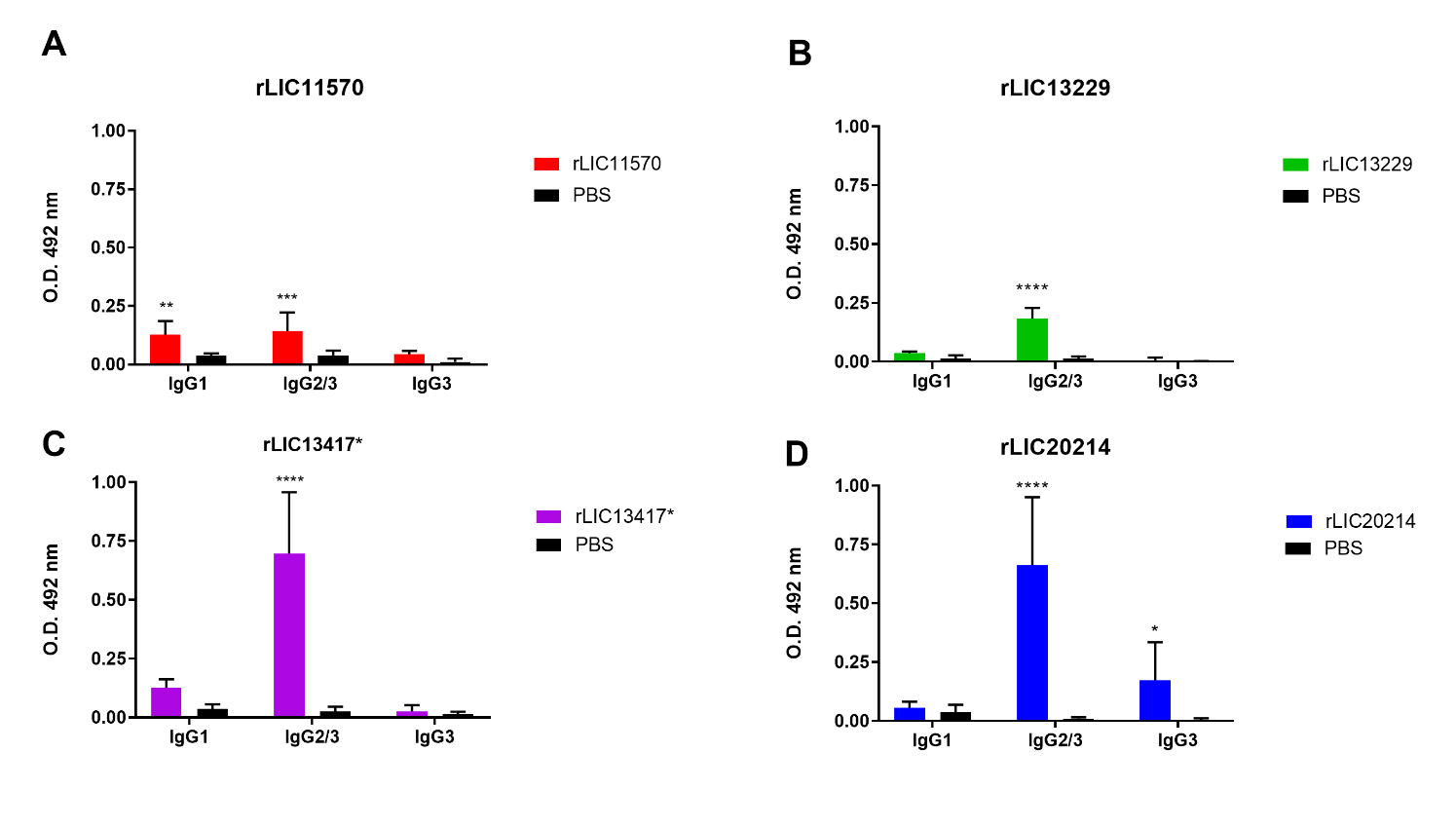


**Figure S5. Determining the anti-rβb-OMPs IgG subclass by ELISA.** Analysis of the IgG subclasses was performed on serum samples collected from surviving animals. The recombinant βb-OMPs rLIC11570 (GspD), rLIC13229 (AlgE), rLIC13417* (AlgE) and rLIC20214 (TBDR) were used as antigens (A-D, respectively). Levels of each subclass were compared within each group and against the PBS/Alhydrogel control. The results are presented as the mean absorbance ± standard deviation, calculated from individual serum samples assayed in triplicate from two independent experiments. Significance was determined by the analysis of variance (Bonferroni multiple comparison): *****P* < 0.0001, ****P* < 0.001, ***P* < 0.01 and **P* < 0.05 indicates significant differences. OD_492_, optical density at 492 nm.
